# Evaluating and strengthening the health system of Curaҫao to improve its performance for future outbreaks of vector-borne diseases

**DOI:** 10.1186/s13071-021-05011-x

**Published:** 2021-09-26

**Authors:** Vaitiare Mulderij-Jansen, Izzy Gerstenbluth, Ashley Duits, Adriana Tami, Ajay Bailey

**Affiliations:** 1 Department of Medical Microbiology and Infection Prevention, University of Groningen, University Medical Center Groningen, Groningen, The Netherlands; 2 International Development Studies, Department of Human Geography and Spatial Planning, Faculty of Geosciences, Utrecht University, Utrecht, The Netherlands; 3 Department of Epidemiology, Curaçao Biomedical & Health Research Institute, Willemstad, Curaçao; 4 Epidemiology and Research Unit, Ministry of Health Environment and Nature of Curaçao, Willemstad, Curaçao; 5 Red Cross Blood Bank Foundation, Willemstad, Curaçao; 6 Department of Immunology, Curaçao Biomedical & Health Research Institute, Willemstad, Curaçao

**Keywords:** Caribbean region, Dengue, Zika virus, Chikungunya virus, Mosquito control, Communication, Developing countries

## Abstract

**Background:**

Vector-borne diseases (VBDs) such as dengue, chikungunya, and Zika pose a significant challenge to health systems in countries they affect, especially countries with less developed healthcare systems. Therefore, countries are encouraged to work towards more resilient health systems. This qualitative study aims to examine the performance of the health system of the Dutch Caribbean island of Curaҫao regarding the prevention and control of VBDs in the last decade by using the WHO health system building blocks.

**Methods:**

From November 2018 to December 2020, a multi-method qualitative study was performed in Curaçao, applying content analysis of documents (*n* = 50), five focus group discussions (*n* = 30), interviews with experts (*n* = 11) and 15 observation sessions. The study was designed based on the WHO framework: health system building blocks. Two cycles of inductive and deductive coding were employed, and Nvivo software was used to analyse the data.

**Results:**

This study’s data highlighted the challenges (e.g. insufficient oversight, coordination, leadership skills, structure and communication) that the departments of the health system of Curaҫao faced during the last three epidemics of VBDs (2010–2020). Furthermore, low levels of collaboration between governmental and non-governmental organisations (e.g. semi-governmental and private laboratories) and insufficient capacity building to improve skills (e.g. entomological, surveillance skills) were also observed. Lastly, we observed how bottlenecks in one building block negatively influenced other building blocks (e.g. inadequate leadership/governance obstructed the workforce's performance).

**Conclusions:**

This study uncovers potential organisational bottlenecks that have affected the performance of the health system of Curaҫao negatively. We recommend starting with the reinforcement of oversight of the integrated vector management programme to ensure the development, implementation and evaluation of related legislation, policies and interventions. Also, we recommend evaluating and reforming the existing administrative and organisational structure of the health system by considering the cultural style, challenges and barriers of the current health system. More efforts are needed to improve the documentation of agreements, recruitment and evaluation of the workforce's performance. Based on our findings, we conceptualised actions to strengthen the health system's building blocks to improve its performance for future outbreaks of infectious diseases.

**Graphical abstract:**

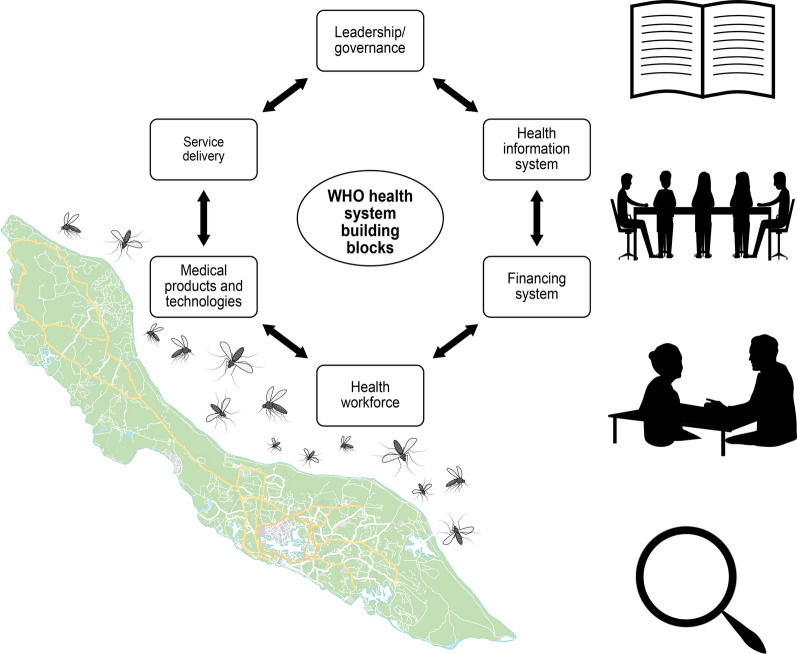

**Supplementary Information:**

The online version contains supplementary material available at 10.1186/s13071-021-05011-x.

## Background

Dengue (DENV), chikungunya (CHIKV) and Zika (ZIKV) viruses are responsible for significant epidemics worldwide [1]. In Curaҫao, dengue is endemic with the co-circulation of the four viral serotypes (DENV-1 to -4) [2]. CHIKV and ZIKV caused an epidemic in 2014–2015 and 2016–2017, respectively [3]. In the Caribbean, these vector-borne diseases (VBDs) are transmitted to humans by the bite of infected *Aedes aegypti* female mosquitoes [4]. Preventing or reducing DENV, CHIKV and ZIKV transmission depends entirely on controlling the mosquito population or interruption of human-vector contact. Factors that influence the rapid expansion of these VBDs are climate change, population growth, urbanisation, international travel and trade, lacking vector control infrastructure/services and less developed health systems [5]. VBDs pose substantial challenges to health systems in the countries they affect, especially resource-limited countries or Small Island Developing States (SIDS).

In the last decade, much attention has been paid to strengthening health systems because there has been growing acknowledgement that a less developed health system is one of the main obstacles to overcome to achieve successful and sustainable public health interventions [6]. The World Health Organisation (WHO) has developed a framework called “Health System Building Blocks” that aims to promote a common understanding of what a health system is and what constitutes health system strengthening [7]. These building blocks define the health system's desirable attributes and offer a mechanism to recognise bottlenecks in structure and performance. A health system consists of all organisations, people and actions whose primary intention is to promote, restore or maintain health [7].

In the WHO framework, a health system is conceptualised as consisting of six building blocks: (i) leadership/governance; (ii) health information system; (iii) financing system; (iv) health workforce; (v) medical products/vaccines/technologies; (vi) service delivery as well as process elements (e.g. access, coverage, quality and safety) and outcomes [8]. According to the WHO, the leadership/governance and the health information systems provide the basis for policy and regulation of all the other health system blocks [8]. Leadership/governance involves ensuring a strategic policy framework, adequate oversight, attention to system design and accountability. An efficient health information system ensures the production, analysis, dissemination and use of reliable and timely information [7]. A good financing system raises adequate funds so that people can use the needed services and are protected from impoverishment [7]. A well-performing health workforce works in responsive, fair and efficient ways to achieve the best health outcomes possible, given available resources and circumstances [7]. A well-functioning health system ensures equitable access to essential medical products and technologies and ensures quality, safety, efficacy and cost-effectiveness. Good service deliveries deliver efficient, reliable, personal and non-personal health interventions to those in need, wherever and whenever care is needed, with minimum waste of resources [7]. Health system strengthening means improving the six building blocks mentioned above and managing their interactions in ways that achieve more equitable and sustained improvements across health services and health outcomes. Both technical and political knowledge and action are required [7].

The WHO framework is valuable because it provides a common language among experts and a good discourse structure on health system affairs [9]. However, for applied research, it needs to be adapted and made context-specific [10]. It has also been argued that the mechanical segmentation of effects by the WHO building blocks, without recognition of their interactions, inhibits the system's understanding [10]. Despite the shortcomings, the WHO building block framework has become the framework most often used to strengthen health systems. It has been used to determine the overall performance of public healthcare facilities [11], implications of health sector reforms [12] and baseline status of health facilities [13]. Furthermore, it has been used to understand the impact of interventions or programmes on the health system in the field of HIV/AIDS [14], malaria [15], measles and polio [10].

The recent epidemics of VBDs in Curaҫao highlight the need to evaluate the health system's performance. This qualitative study aims to examine the performance of the health system of Curaҫao regarding the prevention and control of VBDs in the last decade by using the WHO health system building blocks. This aim will be addressed by assessing and evaluating the structure, organisation, functions, processes and actions performed by the health system concerning the prevention and control of VBDs. The gained knowledge will be used to conceptualise actions to improve the performance of the health system. This study's findings highlight policy and implementation problems worthy of attention and suggest potential solutions to health system bottlenecks. Also, our findings may help better understand the interactions between the building blocks. This knowledge will lead to strengthening of the health system of Curaҫao for future epidemics of not only VBDs but also other infectious diseases (e.g. COVID-19). Furthermore, other SIDS can also benefit from our results and recommendations.

## Method

### Study design

This multi-method qualitative study is based on the interpretive paradigm, and it was performed from November 2018 to December 2020. The study was designed based on the theoretical framework of the WHO health system building blocks. The mentioned framework was applied at the time of data collection, analysis and result interpretation. A combination of different qualitative research methods was used. First, content analysis of governmental documents was carried out to understand the governmental structure in more depth and to gain insights into all that has been written (e.g. protocols, laws and action plans) on prevention and control of VBDs in Curaҫao. Information drawn from the content analysis was used to design topic guides for focus group discussions (FGDs) and interviews with experts. Second, FGDs and interviews with experts were performed to gain insights into motivations, practices, institutional issues, experiences and perceptions of the health system's workforce. Information drawn from the FGDs and interviews with experts was used to validate and support information drawn from the content analysis. Third, observations (e.g. observe house inspections performed by the vector control unit) were conducted to understand further institutional bottlenecks and the working procedure of the health system's workforce. The observations were used to validate and support the information drawn from the content analysis, FGDs and interviews with experts.

### Study site

In October 2010, the Netherlands Antilles (the Netherlands Antilles was a constituent country of the Kingdom of the Netherlands and consisted of several islands located in the Caribbean Sea) was dissolved, and Curaҫao became an autonomous country within the Kingdom of the Netherlands. The island has a surface area of 444 km^2^ and a moderate tropical climate with two seasons (rainy and dry season), with an average temperature of 25–28 °C. According to the Central Bureau of Statistics of Curaçao, the estimated population was approximately 158,665 inhabitants on 1 January 2019 [16]. There are different ethnic backgrounds, with an Afro-Caribbean majority and minorities such as Dutch, French, Latin American, and South and East Asian, Portuguese and Levantine people [3]. Papiamentu, Dutch and English are the official languages of Curaçao, with Spanish also being widely spoken.

### Study population

A total of five FGDs (*n* = 30) were conducted with professionals that have worked for the Ministry of Health, Environment and Nature of Curaҫao (MoHEN). Three of the five FGDs were conducted with professionals who have worked in the vector control unit (VCU), one was held with previous ministers of health and one with health professionals that who worked in policy, communication, vector control and the Epidemiology & Research Unit (ERU). Interviews were conducted with 11 experts. The characteristics of the study population are presented in Additional file 1: Table S1. The numbers of FGDs and interviews with experts were determined after data saturation, where information starts to repeat itself, had been reached. Study participants were recruited using key informants such as heads of departments, and experts were chosen based on their characteristics such as experience and knowledge. The study sample consists of professionals from all six WHO building blocks analysed in this paper.

### Data collection

#### Content analysis

Downe-Wambolt defines content analysis as “a research method that provides a systematic and objective means to make valid inferences from verbal, visual, or written data in order to describe and quantify specific phenomena” [17]. In health sciences, content analysis has been employed to achieve different aims, for example, to evaluate health campaigns [18] or to determine the main challenges of processes related to health service [19]. In this study, the content analysis of documents (e.g. reports, protocols, images, radio and television spots) was conducted first because it provided data on the context within which the study participants and departments had operated during the VBD epidemics. Documents bear witness to past events (e.g. conducted vector control interventions), provide a means of tracking change and development and highlight the conditions that influence the phenomenon under investigation [20]. The following inclusion criteria were applied: (i) the document was original, (ii) it was related to DENV, CHIKV and ZIKV, and (iii) it was developed and used by governmental officials within the 1 January 2010–28 February 2020 time frame. This time frame included the last massive outbreak of DENV infection in 2010/2011 and the CHIKV and ZIKV infection epidemics in 2014–2015 and 2016–2017, respectively. The following exclusion criteria were applied to the documents: (i) the document had no publication date and author details; (ii) it was not related to governmental actions regarding prevention and control of DENV, CHIKV and ZIKV. After correcting for irrelevant or repetitive documents, a total of 50 items were included in the content analysis. An overview of the items used in the content analysis of documents is presented in Additional file 2: Table S2.

#### Focus group discussions and interviews with experts

Five FGDs and 11 interviews with experts were conducted in the second phase of data collection. The topic guides were semi-structured to cover the six health system building blocks and the health system bottlenecks that were drawn from the content analysis of documents (Additional file 3: Text S1, Additional file 4: Text S2, Additional file 5: Text S3 Additional file 6: Text S4, Additional file 7; Text S5: Additional file 8: Text S6). The topic guides were piloted and adjusted before the data collection. The FGDs and interviews were conducted in Papiamentu or Dutch, recorded, and transcribed.

#### Observations

Based on the information collected during the interviews and the FGDs with health professionals, we conducted five follow-up observation sessions during inspections of the VCU to verify reported data related to prevention and control measures and their related challenges. The VCU organised six training sessions regarding mosquito species in Curaҫao and prevention and control of mosquito-borne diseases. We participated in these training sessions to determine the knowledge and entomological skills of the VCU workforce. Also, presented challenges of the workforce were recorded. Furthermore, we participated in four meetings organised by the VCU, the Department of Risk Communication (RC) and ERU to observe how departments collaborate to work on international health regulations, RC, and prevention and control of VBDs. The observations were recorded as notes and pictures. One observer and one note-taker conducted the observation sessions. The collected information was compared at the end of each observation session to identify potential discrepancies. Additional observation sessions were conducted to clarify these differences and reach common conclusions.

### Data analysis

The data coming from FGDs and interviews with experts were analysed using different codes, which refer to an idea, issue, topic or opinion evident in the data. Some of the codes were raised by the study participants themselves (inductive). In contrast, others were prompted by the interviewers using topics in the interview guide that were derived from literature and existing theories (deductive). We employed two cycles of inductive and deductive coding. In the first cycle, codes were used when analysing FGDs and interviews with experts. These codes were assigned to ten categories, which were analysed in the second cycle of analysis. The following categories were identified: (i) leadership/governance, (ii) financing system, (iii) medical products and technologies, (iv) health information system, (v) workforce, (vi) service delivery, (vii) trust, (viii) prevention, (ix) evaluation and (x) recommendation (Additional file 9: Table S3). The analysis of the content of documents and observations was as follows: first, all text was read several times for familiarisation with the content. Second, data from the content analysis of documents and observations were coded using the coding list used for the FGDs and interviews with experts. The same coding list was used because it offered the opportunity to link and compare the data. This data analysis method facilitated the data interpretation to elicit meaning, compare data, gain understanding and develop empirical knowledge. The data were analysed using Nvivo (version 12 Pro). All quotes in the current research were transliterated to keep the context intact.

## Results

The results are presented here with the following key themes: leadership/governance, health information system, financing system, health workforce, medical products and technologies, and service delivery. Each paragraph starts with a structural bottleneck and ends with its implication for the health system's performance.

### Leadership/governance

**Fig. 1:**
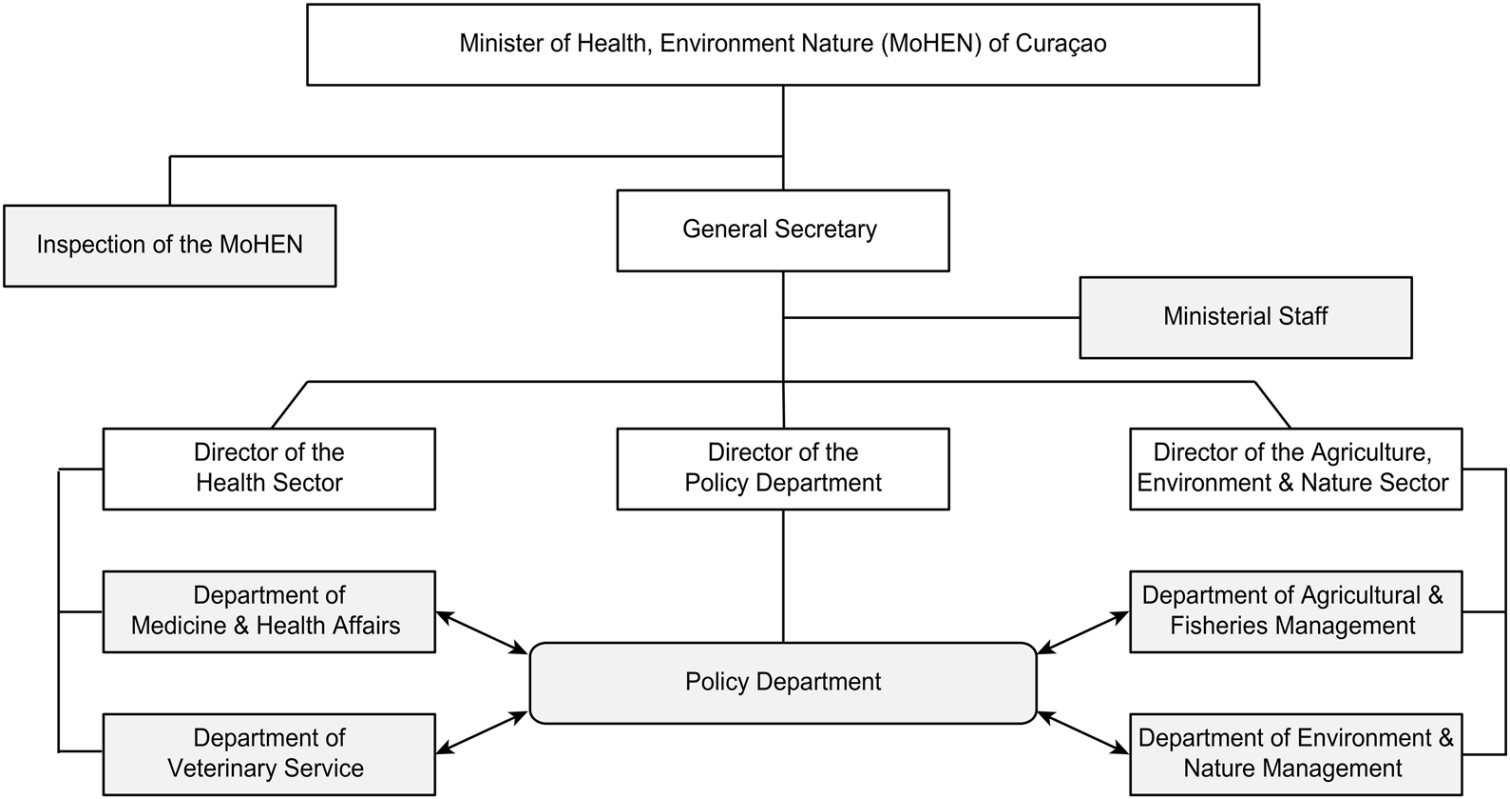
Organisational chart of the Ministry of Health, Environment and Nature of Curaçao. Adapted from Curaçao: Business plan, Ministry of Health, Environment and Nature (p. 10), by MoHEN, 2011. Adapted with permission

It is essential to understand the organisational structure of the MoHEN to understand each department's responsibilities regarding the prevention and control of VBDs. The organisational structure of the MoHEN is illustrated in Fig. 1. Regarding the prevention and control of zoonotic diseases, the Policy Department is responsible for making laws for the Department of Technical Hygiene and Care (THZ). The THZ, which is part of the Department of Medicine and Health Affairs, is responsible for implementing and enforcing the laws (Curaçao: Business plan, Ministry of Health, Environment and Nature, pg: 10–11). Different experts have confirmed the mentioned task division. However, at the same time, they indicated that the mentioned departments do not perform their tasks as is documented. Also, they indicated that the collaboration between the Policy Department and other departments of the MoHEN is minimal. The following quote illustrates this issue:

*Moderator: Okay, there is no law or protocol?*

*Female 1: No, look.*

*Male 2: We met a few times.*

*Female 1: Yes, but with the dengue team (microbiologist, head of the THZ, entomologist, epidemiologist, medical doctors, communication expert).*

*Male 2: The team never received feedback. There was no feedback.*

*Female 1: We asked for the protocol every time. Yes.*

*Male 1: Look, the process to develop a law/protocol is not clear for us (policymakers working for the Policy Department) either. We complained many times about this issue within the department. The problem remains the same. Again the departments that work in the field know the content of their work; they are the experts, not someone that sits in the Policy Department. The policymakers working for the Policy Department need to guide the departments to develop their protocols/law/guidelines, but the content needs to come from the departments, and this is not happening.*

FGD with health professionals working for the MoHEN

**Fig. 2:**
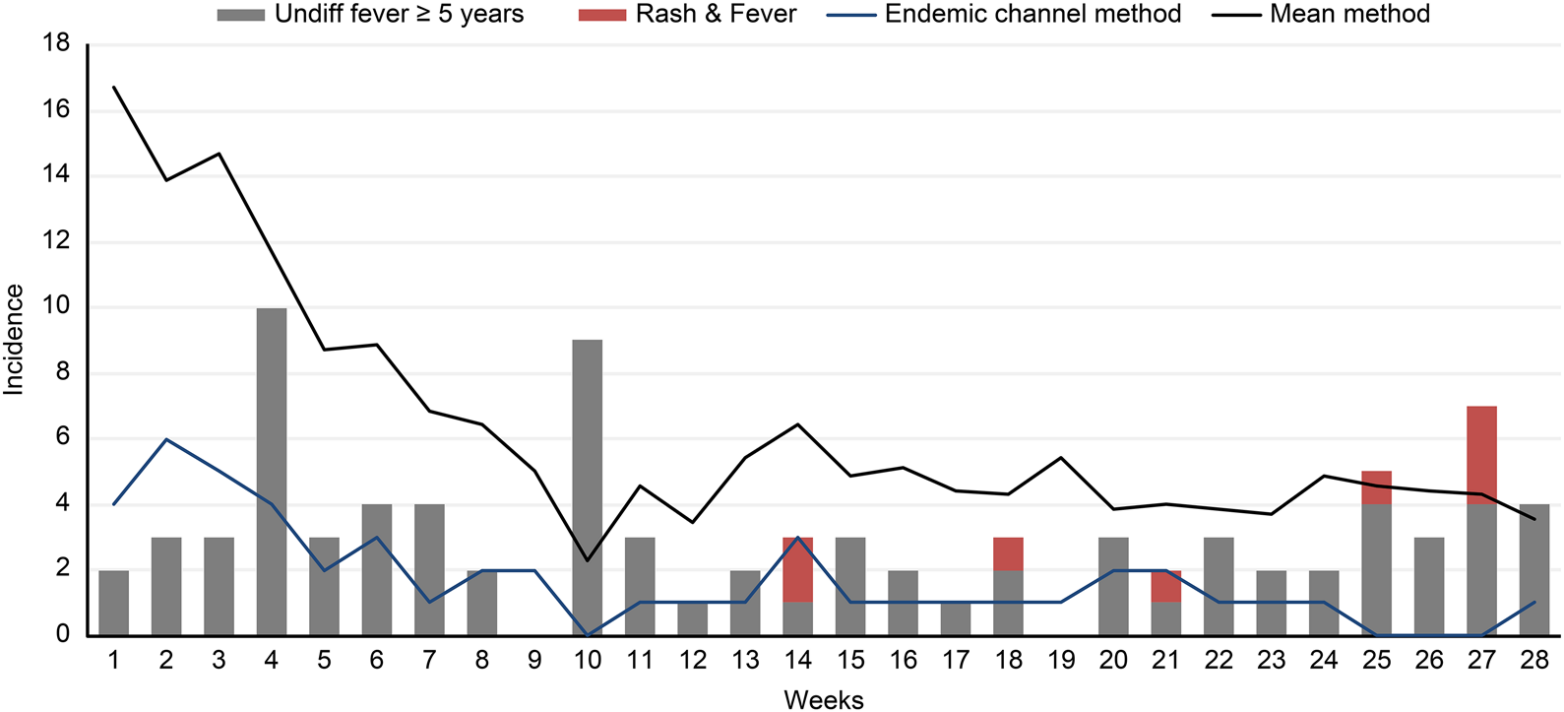
Comparison of two cut-off values to detect outbreak/epidemics. Reprinted from Syndromic Surveillance 2020 by the Department of Epidemiology and Research Unit Curaҫao, 2020. Note: Both methods use fever and rash as a proxy for DENV, CHIKV and ZIKV infection

Insufficient collaboration causes issues such as the development of laws/protocols/guidelines for preventing and controlling VBDs to fall short on certain ends. This issue has been confirmed by notes derived from meetings organised by departments of the Department of Medicine and Health Affairs. The following observation note illustrates this issue:

The RC department organised a meeting to discuss a health promotion campaign for mosquito-borne diseases in Curaҫao. The meeting included professionals from different departments, such as the VCU and the ERU. When the topic “collaboration” was introduced, one of the health professionals asked permission to express her concern.

She was not happy and showed signs of frustration. For example, she tapped her fingers and scratched the back of her head. When she got permission to speak, she said the following: We have a Policy Department, but the professionals working for this department do not know what they need to do. The "real" professionals are working for the departments that are responsible for the implementation of the law/policy. It does not make sense. There is no collaboration between the Policy Department and the departments that are responsible for the implementation of the law. That is why there is no law.

The other health professionals agreed with her statement by saying: “You are right” or “Exactly” or “I could not agree more.”

At that moment, everybody started to talk, and the moderator of the meeting had no control over the invited health professionals. It looked like everybody was not happy with the current collaboration.

Observation notes of a meeting held on October 22, 2018

Data drawn from FGDs and interviews with experts revealed that collaboration between departments is lacking because the workforce's work procedure is not documented in any policy or law. Two evaluation reports confirmed the fact that there is no overall strategic policy framework that guides departments during epidemics of infectious diseases. There is a range of laws, technical briefs and plans that address the prevention and control of VBDs; however, these laws/plans have their shortcomings. There is a law on infectious diseases (verordering bestrijding van besmettelijk ziekten P.B.1921, no 61) that has been used to guide the prevention and control of new VBDs. However, its usability has been questioned in two evaluation reports and by different experts. The evaluation reports and interviewed experts indicated that the law is outdated since it was created in 1921. Also, the law does not contain information concerning the work procedure and responsibilities of needed professionals, departments and organisations to prevent and control VBDs. These shortcomings obstruct collaboration, oversight and accountability within the health system of Curaҫao.

A regulatory law on pesticides (Landsverordering bestrijdingsmiddelen P.B. 1961, no 116) was introduced in 1961 by the government of the Netherlands Antilles. This law addresses rules for importing and using insecticides; however, it does not address the safe use of insecticides and their application in nature. Data drawn from an evaluation report written by two external medical entomologists in 2016 confirmed this issue. More recently, a local medical entomologist explained that since there is no regulation on the safe use of insecticides and application in nature, private and governmental pest controllers can use insecticides that negatively affect our environment without prosecution. The workforce of the VCU stated that the reported lack of regulations concerning the safe use of insecticides and their application in nature weakens the authority and control mechanism of the MoHEN. Both external and local medical entomologists reported that the existing law on pesticides has not been updated or revised since 1961 by the government of the Netherlands Antilles or Curaҫao.

Many participants reported that the organisational structure of the MoHEN introduced after October 2010 (thus, when the Netherlands Antilles was dissolved and Curaҫao became an autonomous country within the Kingdom of the Netherlands) was just copied from that of the Netherlands. This process took place without taking the workforce's capacity or cultural, financial and social factors of Curaҫao into account. All the interviewed ministers of health and most of the interviewed experts indicated that the organisational structure of the MoHEN is weak and observed that its deterioration would continue if the system is not adequately evaluated and adjusted. The following quote illustrates this issue:

*The MoHEN has deteriorated in the last years. The deterioration started after October 10th, 2010. Especially the VCU, which is part of the Department of Technical Hygiene and Care. A large group of personnel retired, some passed away, and some left. In other words, the work of the VCU is non-existent. The ERU follow notifications of PAHO or WHO, but the health system cannot act on the health threats proactively. This was the problem during the epidemic of chikungunya and Zika in Curaҫao.”*

Stephan, policy-maker/general practitioner

Other collected data related to this theme revealed that this organisational problem causes issues such as inadequate tasks and power division. Also, it negatively affects how decisions are made and activities are carried out.

We found that lack of documentation and collaboration between departments obstructs policymaking and the health system management. Experts indicated that most of the agreements concerning VBDs were made verbally and were not documented during the epidemics. Only one document (Integrated Management Strategy for Dengue Prevention and Control in Curaҫao, 2012) was found that contains agreements made by a multidisciplinary team regarding the prevention and control of DENV infection. We found that the lack of documentation leads to delays in key processes or holds up projects, restricts collaboration and causes loss of data, communication gaps, uninformed decisions and lack of transparency.

Experts working for the ERU, VCU and the Policy Department indicated that there was a plan (Project plan: Breeding site elimination and cleaning action Curaҫao, November 2014) that addressed the prevention and control of the *Ae. aegypti* mosquitoes during the CHIKV infection epidemic. However, this plan was made without collaboration and was not shared within the health system. The performed document analysis verified the existence of this plan, and the interviews conducted with experts and FGDs highlighted the fact that only one participant, who developed the plan, was aware of the plan's content. The mentioned plan only focuses on a public health campaign conducted during the CHIKV infection epidemic. It describes a part of the task of the VCU, Selikor (an organisation assigned to perform waste management) and a designated organisation that focussed on clearing streets. However, relevant departments/organisations/professionals such as the ERU, general practitioners (GPs) and laboratories are not mentioned, and an extended description of the multidisciplinary collaboration is not present in the plan. The reported lack of documentation and collaboration causes frustration among co-workers. The following quote illustrates this issue:

*Male 1: But explain this to me, so you have made a plan/protocol for the VCU?*

*Male 3: Yes.*

*Male 1: For vector control?*

*Male 3: Yes.*

*Male 1: Where is it now?*

*Male 3: I do not know; the general secretary has it now.*

*Male 1: But you can not say that. The management team needs to approve it first. Did you discuss this with the management team?*

*Male 3: I do not know. Who is part of the management team?*

*Male 1: The general secretary (SG), director of the sectors (SD) and the director of the Policy Department are members of the management team. However, nobody else from the MoHEN (e.g. epidemiology) has received this plan?*

*Male 3: I do not know.*

*Female 1: I am glad that this group discussion is being recorded. In front of all my colleagues here, he said that he made a protocol for the VCU. A few minutes ago, we spoke about the importance of collaboration and that we need to work together, and look, he worked again on his own.*

*Male 1: He made it on its own, again! Moreover, he is implementing his protocol.*

*Moderator: So this is an excellent example?*

*Male 1: Yes.*

FGD with health professionals working for the MoHEN

The quote above illustrates how separated from each other, in a siloed manner, the employees of the MoHEN work. In addition, interviewed experts indicated that leadership skills and knowledge regarding VBDs among the personnel with leadership functions were insufficient. This issue obstructs guidance and collaboration for developing protocols for the prevention and control of VBDs. For example, obtained data collected from documents and interviews with experts highlighted the fact that policies/protocols/plans (e.g. surveillance of microcephaly or pregnant women) were also lacking during the Zika epidemic.

### Health information system

Data drawn from this qualitative study highlighted the following challenges regarding the collection, analysis and dissemination of information concerning VBDs within and outside the health system of Curaҫao.

#### Challenges associated with entomological practices

Experts indicated that the VCU, which is responsible for collecting information related to mosquitoes and their breeding sites, has been facing challenges to recruit and maintain professionals with the capacity to collect entomological data. This challenge, in turn, obstructs the data collection of local mosquito fauna. A report written by two external medical entomologists confirmed this statement. Additional file 10: Table S4 presents published data concerning mosquito species of Curaҫao. Both the report and some experts agreed that information concerning local mosquito fauna is scarce and old (from the late 1940s). A local medical entomologist elaborated on this topic and reported six more species (i.e. *Aedes infirmatus*, *Aedes sollicitans*, *Aedes triseriatus*, *Aedes vexans*, *Coquillettidia perturbans*, *Aedes condolescens*) that need to be added to the list. However, the presence of these additional species needs to be verified. The workforce of the VCU also confirmed this issue. They stated that the surveillance of mosquito species is non-existent. The following quote illustrates this issue in more depth.

*Moderator: Okay, let's continue with the next topic. We spoke about the problem concerning documentation. The steps that need to be taken are not clear. We spoke about the challenges that departments of the government had during the epidemics. We spoke about laws and structure. Let us talk about surveillance, surveillance of the vector.*

*Can you describe how surveillance of mosquitoes was performed during the epidemics? And currently? Do we monitor mosquito species present in Curaҫao, and do we test the mosquitoes to check for viruses?*

*Male 4: What do you mean? The control that we are performing now?*

*Moderator: No, I am talking about the surveillance of the vector, not vector control. For example, the ERU is responsible for the surveillance of cases of VBDs, and the VCU is responsible for the surveillance of the mosquitoes.*

*Male 4: Look, we had a surveillance system, but currently, we are not monitoring mosquitoes because we do not have the means to do it. We need to create a structure for the surveillance of mosquitoes to have continuity. I mentioned this in the policy that I made for the VCU.*

*Moderator: Thus, currently, there is no surveillance of mosquitoes?*

*Male 4: No, there is no surveillance of mosquitoes. We are using old information and data that you (PhD students) are currently collecting.*

FGD with health professionals working for the MoHEN.

Since there is no ongoing surveillance of mosquitoes, there is no monitoring of the insecticide resistance status of *Aedes* populations. This leads to minimal dissemination of information concerning the vectors of DENV, CHIKV and ZIKV within the health system.

#### The reliability of collected entomological data

Some experts have questioned the analysis, interpretation and entomological data usage. According to an evaluation report, the ERU analyses collected data concerning larvae and pupae to determine entomological indices (e.g. household index, container index) for the VCU. Interviewed experts working for the ERU reported this written task division and indicated that the mentioned data analysis happened once. The data were collected in 2015 (not the entire year) by the VCU and analysed in 2016. Experts working for the ERU reported that the data analysis was obstructed because the collected data were unreliable. The data were collected by a workforce with limited capacity concerning entomological field techniques. Data drawn from the FGDs and interviews with experts revealed that the lack of entomological information affects the performance of VCU negatively because changes in geographical distribution and density of the vector are not taken into account to guide vector control strategies. Also, these missing data obstruct the evaluation and adjustment of vector control strategies, in turn negatively affecting decision-making processes.

#### Challenges associated with surveillance systems

Some experts have also questioned the surveillance systems (laboratory and syndromic surveillance systems) that the ERU uses to detect the presence and activity of VBDs in Curaҫao. Data drawn from this qualitative study highlighted the following two critical bottlenecks which obstruct the surveillance of cases of VBDs:

1. Delays in disseminating information: The time frame to report the laboratory-confirmed cases to the ERU was greatly extended in the last 5 years. Delays in disseminating information concerning laboratory-confirmed cases could have caused delays in detecting the onset of VBDs in Curaҫao. The following quote illustrates this issue:
  *Before 2008, the dissemination of information concerning laboratory-confirmed cases was conducted in a timely fashion. However, after 2008 a deterioration in the dissemination of laboratory information was observed. This is possibly caused by the competition between laboratories, lack of guidance and policy concerning the surveillance of VBDs. So, the information that I am receiving from the laboratory cannot be used to detect epidemics of VBDs because it takes months before the information is reported. We are walking behind the facts.*
  Sarah, epidemiologist

2. The surveillance system's sensitivity: Several experts have stated that prevention and control actions to combat the CHIKV infection epidemic started late. One expert indicated that the syndromic surveillance system was not sensitive enough to detect the CHIKV infection epidemic's onset. The epidemiologist responsible for the surveillance system during the CHIKV infection epidemic concluded in late 2014 that the method (mean incidence) used to identify the epidemic of CHIKV infection was less sensitive than the Endemic Channel method. The Endemic Channel method was introduced during the CHIKV epidemic (beginning 2015); this method uses the previous 7 years' surveillance data (e.g. fever as a proxy for some VBDs) to determine three threshold levels. These cut-offs would allow the epidemiologist to identify how the current incidence would relate to past data, indicating if the observed incidence is below what is expected (success), as expected (safe) or higher than expected (alert). The Endemic Channel method is more suitable to detecting epidemics in small countries with small data sets. This statement has been confirmed with the information illustrated in Fig. 2.

Despite the information from PAHO/WHO alerting all countries of the Americas, these two bottlenecks could be possible explanations for the reported delay in detecting the onset of the CHIKV infection epidemic. The reported delay in detecting the onset of the CHIKV infection epidemic might have obstructed the response of the ERU to inform needed stakeholders (e.g. GPs, VCU, communication department, laboratory, etc.) to start actions related to the prevention and control of VBDs.

#### Challenges associated with disseminating information

All interviewed experts indicated that the dissemination of information within the health system and to the community or other stakeholders was minimal during the last three VBD epidemics, especially during the CHIKV infection epidemic. For example, GPs, an important stakeholder group, have stated that the government's communication needs to be improved because limited instructions and updates were offered during the epidemics. Also, it has been stated that information regarding VBDs did not reach every GP. The following quotes illustrate the mentioned communication problems:

*GP: I think that the flowchart of information needs to be improved. Look, the national epidemiologist reaches a specific group of GPs that are members of the association of GPs. It is always the same GPs that are present during presentations and meetings organised by the association. For example, when you go to meetings organised by the association, about 40 of the 100 GPs are present. Thus, there is a group that you are not reaching.*

*Interviewer: That is a piece of useful information.*

*GP: For example, in most cases, GPs who speak Spanish are not present: thus, they are a group that is difficult to reach. Also, with e-mails.*

*Interviewer: Do you notice that fewer Spanish-speaking GPs attend the meetings?*

*GP: Yes, they are doing their best to change how they share information. The information is shared in Papiamento or English. However, English can be challenging to understand.*

Sol, geriatrician

*It is interesting because many people were tested without no valid reason, like old females. Why? Was the protocol not clear for the GPs?*

Sarah, epidemiologist

*GP: In the case of CHIKV infection, I had the idea that the GPs were confused; the recommendations were not concrete, testing or no testing? I think the capacity to test was limited. I am not sure about the problem, but the recommendations were not clear. The community was sick, and it was challenging to contact The MoHEN. I called a few times to ask for advice.*

Sol, geriatrician

Communication problems within and outside the health system have been discussed in different documents; for example, in an evaluation report, written by the “Court of Audit Curaҫao”, entitled “Report Chikungunya”, and observation notes of meetings organised by the workforce of the health system. The evaluation report mentioned above confirmed the reported lack of communication between stakeholders, including GPs, and highlighted that the absence of a communication strategy plan obstructed communication between the stakeholders during the epidemic of CHIKV infection.

### Financing system

The method used to raise funds for the departments responsible for preventing and controlling VBDs has been discussed in five documents (two evaluation reports, the budget of the MoHEN 2019–2022 and two observation notes), FGDs and interviews with experts. The government of Curaҫao mainly generates funds for its ministries through taxes. There is no specific fund destined for the MoHEN. The MoHEN develops a budget each year and submits its financial request to the government; the budget is reviewed to set priorities. We could not locate and review the assigned budget to prevent and control DENV, CHIKV and ZIKV infection epidemics. Lack of documentation could explain why we did not get an overview of the budget and the expenses made in the last years.

A budget made for 2019–2022 was obtained, and it indicates funds that are meant to be available for the prevention and control of VBDs. For example, 25,000 NAF (Antillian Guilders) (± 13.740 USD, 1 USD = 1.82 NAF) was available for detection and control of pests (e.g. the use of insecticides or pesticides), 50,000 NAF (± 27,470 USD) for health promotion, including RC, 50,000 NAF for the training of the workforce of the THZ, 25,000 NAF for purchase of machines and 7500 NAF (± 4120 USD) for other materials (e.g. uniforms, gloves, etc.). These amounts were budgeted for 2019, 2020, 2021 and 2022, respectively. This budget was presented and discussed during FGDs and interviews with experts; remarkably, most interviewees never saw this budget. Data drawn from these interviews/discussions indicated that a critical bottleneck in the financing system is the manner of making the budget for the departments of the MoHEN. Some experts stated that during the CHIKV infection epidemic, the budget of the departments of MoHEN was made by a government official with authority. The budget was never presented or discussed with the involved departments. The following quote illustrates this issue:

*Male 1: Yes, what he needs to do is talk with the heads of departments every year, to plan, to know what the departments need to function. Ask what they need for the next year? How much personnel is needed? Materials? Talk about these factors and develop a budget. He added all the expenses together, for example, health promotion materials and came with a budget for all departments. He needs to explain how much each department needs for health promotion and give each department their money when it is needed. That is what he needs to do, but this never happened.*

*Male 2: He needs to respect the budget, too.*

*Female 1: Yes, respect it because currently, they are using the budget of one department to cover financial issues of another department.*

FGD with health professionals working for the MoHEN

The reported lack of collaboration in drafting the budget of MoHEN leads to a budget that is not realistic and suitable to cover the expenses of the involved departments during the epidemics. For example, a policymaker indicated that during the ZIKV infection epidemic, 50,000 NAF was available for health promotion; however, the actual expenses were 150,000 NAF (± 82,420 USD). The presented budget for 2019–2022 also confirmed the fact that the budget does not take economic inflation into account because the same amount of money has been budgeted for each year. Besides, both an evaluation report and the majority of interviewed experts indicated that the funds allocated for specific expenses (e.g. working materials, training, etc.) are not available when needed. The workforce of the VCU elaborated further on this topic and stated that due to limited financial support, there were limited materials (e.g. insecticides), personnel and training for personnel during the CHIKV infection epidemic. The following quotes illustrate this issue:

*Male 2: There was no workforce at that moment.*

*Female 2: Workforce, there was no personnel to perform control of mosquitoes.*

*Male 2: We need to blame the government again because there were no inspectors to perform vector control.*

*Female 4: Yes.*

*Moderator: Thus, when the CHIKV infection outbreak started, there were no inspectors to perform vector control?*

*Male 2: No, nobody.*

*Female 4: No.*

*Moderator: Thus, you looked for personnel during the epidemic of CHIKV infection?*

*Male 2: Yes.*

*Female 4: Twenty kids, twenty young people.*

*Female 2: Educated fieldworkers who worked for the MoHEN retired, and new employees were not recruited.*

FGD with the VCU workforce

*Male 1: We need uniforms; my white shirt is almost brown now.*

*Female 3: Our badges expired long ago.*

FGD with the VCU workforce

*Moderator: You are working now with Abate?*

*Male 1: Yes.*

*Moderator: Do you have the possibility to wash your hands after using Abate?*

*Male 1: No.*

*Female 2: That is what he explained.*

*Female 1: We got a hand sanitiser to disinfect our hands.*

*Male 1: No mask or gloves were given. We need to supply ourselves with what we need.*

*Female 1: Nothing was given to us.*

FGD with the VCU workforce

Remarkably, funds are being allocated for the prevention and control of VBDs. However, it is not being used as planned in reality or does not cover the actual expenses. Experts stated that the performance of departments has deteriorated due to the budget retrenchments that happened in the last two decades. No investments were made to maintain the capacity and quality of vector control, research and communication practices. Previous ministers of health have confirmed this issue, and according to one interviewed minister, the government needs to change its approach to deal with the health sector, especially with prevention. The government does not see its expenses as investments to improve the health sector. This mindset leads to more budget retrenchment.

### The health workforce

The MoHEN Business plan (2011–2014) provides information concerning the required formation for a well-functioning unit to prevent and control zoonotic diseases in Curaҫao. This estimated formation has been compared with the available workforce during the epidemics of VBDs (Additional file 11: Table S5). After comparing this information, it can be stated that the departments responsible for the prevention and control of VBDs are understaffed. For example, according to the Business plan, the ERU needs nine full-time employees (FTEs) (Additional file 11: Table S5). However, during the last three epidemics, the ERU consisted of three employees: one medical doctor/epidemiologist (1 FTE) and two other health professionals specialised in epidemiology and public health (2 FTEs).

As shown in Additional file 11: Table S5, and according to an evaluation report (written by two external medical entomologists), the VCU had no head, entomologist or any employee able to identify mosquitoes to species level during epidemics of CHIKV and ZIKV infection. Besides, the VCU field workers had their contracts renewed several times because they have short-term employment contracts. The field workers elaborated further on this topic and stated that the insecurities concerning their contracts reduce their motivation to perform their duties. Also, most of the field workers feel that they are being used and are not valued. The following quotes illustrate concerns of the field workers:

*Male 1: We got more things to do and what happened is that our work got mixed-up. That is the reason why I told my coordinators that they are using us. We, as field workers, are being used. We are here to prevent and control mosquitoes; thus, we need to work only with mosquitoes. We cannot work with all types of vectors because we do not have the education. We need to stand up and say we came here to work with mosquitoes.*

*Moderator: Okay.*

*Male 1: That is what we need to do.*

FGD with the VCU workforce

*Female 1: Look, the field workers that are working for the VCU are not inspectors specialised in vector control. I know that you are recording, but this is the truth. They are not inspectors.*

*Interviewer: I understood that they are not inspectors for vector control, but they have worked with different types of vectors.*

*Female 1: Yes, they are not educated inspectors. They are being used as a shield, and that is the problem. It is criminal because the government creates a false sense of security. That is not okay.*

Elsa, entomologist/policymaker/registered restricted pest controller

As shown by the quotes above, the field workers are called “vector inspectors”; however, according to an evaluation report and interviews with experts, these field workers only received training, limited to 2 weeks, in mosquito prevention and control strategies. Thus, they are not certified to do all types of work related to vector control. Due to limited financial resources, minimal training opportunities were offered to the workforce in the last 10 years. Most of the certified inspectors have retired, and during the epidemics, the VCU only had three certified inspectors. Besides the VCU and the ERU, the department of communication also faced issues related to its workforce. Several interviewed experts indicated that the department of communication employees do not have the education, skills and creativity to develop a strategic communication plan to reach most of the community. Both participants of the FGDs and interviews questioned the manner of recruiting the health system workforce. Some experts have stated that some health professionals have been recruited based on bureaucracy and not on professionalism. Issues mentioned by the workforce have been discussed with the Policy Department and the director of the health sector. The data of this study revealed that these issues are known. However, limited actions have been carried out to improve the workforce's capacity in the last 10 years.

### Medical products and technologies

The treatment of dengue, chikungunya and Zika relies on supportive therapy and symptom relief since no antiviral treatment is available. According to the interviewed GPs, the health system of Curaҫao ensured equitable access to medical products during the CHIKV infection epidemic. Besides medical products, the health system needs to arrange or collaborate with organisations to access the required technologies, such as diagnostics tools. Since laboratories play an essential role in diagnosing these diseases, input and collaboration between laboratories and the health system have been discussed during FGDs and interviews with experts. Several interviewed experts questioned the collaboration between the laboratories and the health system. They stated that the agreements made between MoHEN and laboratories, especially Analytisch Diagnostisch Centrum (ADC) laboratory, the public health laboratory, are not documented. This causes no or delayed reporting of laboratory-confirmed cases of VBDs, no sustainable collaboration and a lack of cost-effectiveness. The following quote illustrates the mentioned issues:

*Male 2: The performance of ADC is not in concordance with the guidelines of a public health laboratory. That is not good.*

*Male 1: The government never defined ADC's role as “the national public health laboratory”. The government just mentioned its role, but it has not been documented.*

*Male 1: They have a severe problem.*

*Female 1: Yes, they have a problem.*

*Male 2: Yes, there is a problem. When I started with my job in 2008,the government gave the national laboratory 2.8 million guilders for public health issues (e.g. to pay laboratory tests).*

*Male 1: Annually.*

*Male 2: Annually? Really?*

*Male 1: Yes.*

*Male 2: But it strange, because if the government needed laboratory tests they needed to pay for the tests again.*

*Male 1: Yes, we [MoHEN] needed to pay again.*

*Male 2: This happened during the dengue outbreak.*

*Male 1: And in the period of Zika, we [MoHEN] paid ADC more money.*

FGD with health professionals working for the MoHEN

An interview with a representative of ADC was conducted to hear their side of the story. However, limited information concerning the collaboration and agreements between the health system and ADC was provided. ADC only indicated that they need to diminish information concerning VBDs when the health system asks for it (e.g. during an epidemic). In contrast, the health system expects their collaboration during the entire year for the surveillance of VBDs.

Larval control was performed using Aquabac™ 200 g granules, guppies (*Poecilia reticulata*) or Abate™. In some cases, kerosene was sprayed in cisterns and sometimes in mangroves. Adult mosquitoes were treated with chemical control measures in response to complaints of a severe nuisance. Adulticiding was performed through hot fogging with Masterline™ Aqua-kontrol 30–30 (a synergised [combining with piperonyl butoxide] synthetic pyrethroid, with permethrin as active substance) with thermal foggers London™ fogger ULV Model 1800E around 5 a.m. or 6 p.m. The interviewed workforce of the VCU indicated that when the CHIKV infection epidemic started, there was insufficient working material, including personal protective equipment and biological and chemical products. The performed observations also confirmed the reported problem with limited resources. The fieldworkers worked with Abate™ without proper protection, such as gloves, safety glasses or adequate clothing, to minimize exposure to the chemical substance. During their fieldwork, they do not have access to water to wash their hands after using the mentioned substance. Also, tools (e.g. tubes, pots, a hand lens, etc.) needed to perform basic entomological research were lacking.

### Service delivery

According to observation notes, the VCU offered the following free services to the community and organisations to prevent and control mosquitoes during the last three epidemics of VBDs: (i) inspect houses of infected locals and their neighbours for mosquito breeding sites, (ii) provide larvicide (e.g. Abate™), (iii) fumigation, (iv) provide guppies (*Poecilia reticulata*) and (v) perform health promotion. Several experts questioned the service delivered by the VCU. They indicated that the service delivered was obstructed by the following factors; insufficient larvicide, educated field workers, entomological field equipment, financial support, guidance and collaboration between governmental and non-governmental organisations. Two evaluation reports have confirmed the mentioned statement.

Furthermore, an evaluation report written by two external entomologists indicated that little scientific literature had been used to update vector control practices in the last decade. A local entomologist confirmed this statement by giving the following example: Abate™ was previously primarily used, but it was replaced with an environmentally friendly substance, Aquabac™. In 2018, Abate™ was re-introduced by the former head of the VCU without taking related consequences (e.g. insecticide resistance) into account. The following quote illustrates this issue:

*Female 1: Yes, we had all that stuff [larvicide]. I introduced a growth hormone. What is its name again?*

*Interviewer: Bti Bacillus something.*

*Female 1: Bacillus thuringiensis israelensis (Bti)(Aquabac™)*

*Interviewer: But Bti was replaced with Abate.*

*Female 1: That is a bad idea. In many aspects, it is a bad idea.*

*Interviewer: But as a toxicologist, he should know that.*

*Female 1: But he does not understand environmental health. Look, please consider this information and take it with you. Maybe you know this already. Abate is still helpful to combat larvae; vectors are predominantly resistant to many substances nowadays. Thus, Abate needs to be your last resource. If you are using it in the long term, you need to plan to switch the substances you are using.*

*Interviewer: Thus, it is like you start treating everybody in the hospital with Colistine?*

*Female 1: Exactly. It's a bad idea, and I told them.*

Elsa, entomologist/policymaker/registered restricted pest controller

Abate™ and Aquabac™ usage within the VCU was also a concern of many experts. They stated that the fieldworkers do not know how to work with larvicide (Abate™) and Aquabac™. The fieldworkers themselves have confirmed this statement. The following quote illustrates this issue:

*Male 1: The employee that hands out Abate; he works in the storeroom; he does not know how to deal with Abate and Bti. He cannot give the correct information. He got some information, and that is what he is using. Just let me asks them [field workers], how much Bti or Abate do you need to treat a small pool? They do not know.*

*Moderator: Thus, what you mean is that the guideline to use Abate and Bti is not clear? Moreover, you are using these products based on feelings?*

*Male 1: Yes.*

*Male 2: Yes. Just like that.*

FGD with the VCU workforce

As shown by the quote above, the lack of knowledge and skills concerning the usage of biological and chemical substances to prevent and control VBDs obstructs the health system's performance.

Lastly, some experts stated that vector control during house inspections has deteriorated because the fieldworker’s work has not been evaluated and supervised by educated inspectors in the last decade. This causes distrust of the VCU and their collected entomological data. Field excursions with the fieldworkers demonstrated unethical behaviour (e.g. falsified house inspections) that diminish trust in the VCU. On the other hand, no access to properties and inhabited houses also obstructs its fieldwork. According to some experts and observation notes, many assigned houses are not inspected since the owners are not at home during the VCU's working hours. Another bottleneck is the planning to perform vector control in a year. Vector control starts at one side of the island and finishes on the other. There are no assigned teams for specified neighbourhoods. Several experts have questioned this approach since it has been shown to be inefficient in preventing and dealing with an epidemic. They also stated that prevention, including RC and mosquito control measures, had been implemented reactively (in response to epidemics, severe nuisances and infected cases) and not from a proactive perspective.

## Discussion

Information concerning health system performance among SIDS is currently lacking in the scientific literature; therefore, research is needed to assess health system bottlenecks to work towards more resilient systems. This qualitative study aimed to examine the performance of the health system of Curaҫao regarding the prevention and control of VBDs in the last decade by using the WHO health system building blocks. The performance of the health system of Curaҫao was negatively affected by insufficient collaboration between governmental and non-governmental organisations (NGOs), leadership skills, coordination, structure, communication within and outside the health system, qualified health workforce and capacity-building. Other SIDS in the Caribbean [21] and the Pacific [22] also struggle with similar organisational bottlenecks as a result of their size, limited resources, geographic dispersion and dependence on foreign markets and financing [23]. Most studies, including the studies mentioned above, focussed on one section of the health system and thus failed to holistically research health system performance. Our study went a step further by using a well-known theoretical framework to highlight the complexity of this matter. We observed how building blocks influence each other. Therefore improving the health system building blocks without recognising their interaction will lead to unsuccessful health system structures and management systems, which will continue to fail to address the existing health challenges [24]. Currently, the health system of Curaҫao faces challenges in controlling COVID-19. Guidelines, oversight, collaboration and communication are still insufficient. Thus, the above-mentioned organisational bottlenecks continue to impose barriers to the health system's performance in the current COVID-19 pandemic.

Our findings also highlight some aspects of the organisational culture of the health system of Curaҫao. We found that the workforce works in a siloed manner to avoid being blamed for mistakes. Also, they believe they must interact with people in ways that will not threaten their employment security because bureaucracy still plays an essential role. The characteristics of avoidance and conventional and approval culture styles are associated with the passive/defensive culture style [25]. Organisations with passive/defensive cultures have many unresolved conflicts, and the workforce often reports low levels of job satisfaction and motivation [26]. These aspects were also observed in this study. This study gave some valuable insights into the culture of the health system of Curaҫao. However, to study this complex mechanism more profoundly, the literature suggests using the Organisational Culture Inventory tool [25].

This study showed how different health system bottlenecks have obstructed the development, approval and implementation of laws/guidelines and interventions, such as the Integrated Vector Management (IVM) programme, in Curaҫao. A common challenge observed in our study and others in implementing the IVM programme is the lack of stakeholder engagement and support [27, 28]. Stakeholder participation in IVM reduces overlap, avoids duplication of activities and saves costs by making better use of existing human and financial resources [29]. All stakeholders need to recognise the significance of the IVM programme and commit to implementing the programme. Otherwise, VBD control will remain fragile in the country. According to the WHO, the IVM programme requires good coordination and oversight to allocate funds and adequate human capacity and to monitor and improve stakeholder engagement [30]. Therefore, we developed an action plan (Table 1) to reinforce oversight of the IVM programme of the health system of Curaҫao. This action plan will provide guidance, create transparency and establish a platform to communicate and collaborate within the health system.

**Table 1:** Action plan to improve the performance of the health system of Curaҫao

	Responsible
**Actions to improve prevention and control strategies concerning VBDs**	
Assign three health professionals with VBD, management and public health experience to coordinate actions related to the prevention and control of VBDs	The management team of the MoHEN
Institute a multidisciplinary team (MDT) including an entomologist, epidemiologist, communication expert, certified vector inspector, sociologists, surveillance systems specialist, laboratory representative (e.g. microbiologist), policymaker, environmental specialist and, if possible, PhD students who have been working in this field Note: The three assigned health professionals are responsible for documenting and disseminating information and coordinating team-related activities. They are members and representatives of the MDT	The three assigned health professionals
Evaluate, adjust and promote laws/policies regarding infectious diseases and vector control (e.g. the existent Integrated Management Strategy for Dengue Prevention and Control in Curaҫao, 2012)	The MDT and policy department
Make a protocol for the usage of biological and chemical control measures, including related safety procedures	The entomologist, certified vector inspector, environmental specialist and policymaker
Evaluate and provide recommendations to improve the working procedure related to housing inspections (e.g. create teams, VCU field workers work under the supervision of certified vector inspectors responsible for specific neighbourhoods/geo zones)	MDT
**Actions to enhance capacity building**	
Make job descriptions for the health system workforce that works in the field of prevention and control of VBDs Note: HR recruits future health professionals based on developed job descriptions	Human resources (HR) and the representatives of the MDT
Seek ways to acquire funds to organise training for the workforce in different aspects (e.g. entomology, surveillance, RC, geographic information system [GIS], equipment calibration, pesticide safety)	The representatives of the MDT
Make use of local published data to make decisions and use the research skills of PhD students to perform the necessary research, for example in the field of social sciences and entomology	The MDT
Perform insecticide resistance and entomological research (e.g. determine the house index container index, Breteau index and pupal index)	The entomologist and VCU
Seek funding to create a basic entomological laboratory to perform basic entomological research	The representatives of the MDT
**Actions to enhance communication and intra- and intersectoral collaboration**	
Seek ways to connect both governmental organisations and NGOs that are needed for prevention and vector control, e.g. Selikor (waste management), Caribbean Research and Management of Biodiversity (CARMABI), Ministry of Traffic, Transport and Urban Planning and Ministry of Social Development, Work and Welfare, Ministry of Education, Science, Culture and Sport and laboratories Note: The representatives of the MDT, management specialist, sociologist and policymaker are responsible for documenting agreements between organisations to enhance accountability and transparency	The representatives of the MDT, management specialist, sociologist and policymaker
**Actions to reform the health system**	
Examine and determine the organisational culture of the health system	Management specialist, sociologist, policymaker and HR
Make a plan to improve the management and culture style of the health system. Provide recommendations to reinforce the organisational structure of the MoHEN	Management specialist, sociologist, policymaker and HR
Evaluate the motivation, job satisfaction and abilities of the workforce of the health system	HR

Qualitative research is often criticised because the findings cannot be generalised and do not prove causality. However, when studying organisational processes, perceptions and experiences of people, qualitative studies provide the opportunity to explore the reasons, motivations and procedures that affect the functioning of the health system. To minimise the mentioned limitations and improve this study's quality, we combined four qualitative research methods to provide a confluence of evidence that breeds credibility. Furthermore, the sample size can be considered small for this type of research. However, this sample is believed to be fairly representative of the distribution of people in the health system's hierarchy; age, gender, experience, education and the participant’s job confirm this.

## Conclusions

Based on our findings, we can conclude that the performance of the health system of Curaҫao was not optimal during the last epidemics of VBDs. Various internal and external factors negatively influenced the performance of the health system in the last decade. There is a strong need for an overall policy, proper job descriptions, a trained health workforce, structural communication and collaboration. Therefore, we recommend: (i) reinforcing the oversight of the IVM programme and administrative structure and (ii) evaluating and reforming the organisational structure of the health system of Curaҫao by using our developed action plan. Undoubtedly other SIDS can also benefit from our action plan since: (i) they cope with similar organisational bottlenecks and (ii) such an action plan covering all health system building blocks to improve health system performance concerning prevention and control of VBDs for SIDS is lacking.

## Supplementary Information

**Additional file 1: Table S1.** Characteristics of the study participants

**Additional file 2: Table S2.** Characteristics of the collected documents

**Additional file 3: Text S1.** Topic guide: FGD with the Vector Control Unit

**Additional file 4: Text S2.** Topic guide FGD with health professionals

**Additional file 5: Text S3.** Topic guide: Interview with general practitioners and geriatrician

**Additional file 6: Text S4.** Topic guide: Interview with alternative medicine practitioners

**Additional file 7: Text S5.** Topic guide: Interview with a laboratory technician

**Additional file 8: Text S6.** Topic guide: FGD with previous ministers of health

**Additional file 9: Table S3.** Coding list

**Additional file 10: Table S4.** List of mosquito species of Curaҫao

**Additional file 11: Table S5.** The workforce during the epidemics compared with the required workforce to perform prevention and control strategies with regards to VBDs

## Data Availability

The data supporting this article's conclusions and recommendations are included in the article, and additional information can be found in the appendix. The raw data will not be made publicly available because participants did not consent to have their full transcript available for the public. Requests to access the data can be sent to Ieneke van der Gun, e-mail address: b.t.f.van.der.gun01@umcg.nl, and Eurydice Martina (project coordinator at Curaçao Biomedical & Health Research Institute), e-mail address: e.martina@cbhri.com.

## Abbreviations

ADC: Analytisch Diagnostisch Centrum
AIDS: Acquired immunodeficiency syndrome
Bti: *Bacillus thuringiensis israelensis*
CHIKV: Chikungunya virus
COVID-19: Coronavirus disease
DENV: Dengue virus
EEE: Eastern equine encephalitis virus
ERU: Epidemiology and Research Unit
FGDs: Focus group discussions
GPs: General practitioners
HIV: Human immunodeficiency virus
IVM: Integrated vector management
MoHEN: Ministry of Health, Environment and Nature
NAF: Antillian Guilders
NGOs: Non-governmental organisations
PAHO: Pan American Health Organization
RC: Risk communication
SD: Director of the sector
SG: General Secretary
SIDS: Small Island Developing States
SLE: Saint Louis encephalitis virus
THZ: Technical hygiene and care
USD: United States dollar
VBDs: Vector-borne diseases
VCU: Vector control unit
VEE: Venezuelan equine encephalitis virus
WHO: World Health Organization
WN: West Nile virus
ZIKV: Zika virus

## References

[1] Beltrán-Silva SL, Chacón-Hernández SS, Moreno-Palacios E, Pereyra-Molina JÁ (2018). Clinical and differential diagnosis: dengue, chikungunya and Zika. Rev Med Hosp Gen Méx.

[2] Elsinga J, van der Veen HT, Gerstenbluth I, Burgerhof JGM, Dijkstra A, Grobusch MP (2017). Community participation in mosquito breeding site control: an interdisciplinary mixed methods study in Curacao. Parasit Vectors.

[3] Mulderij-Jansen V, Elsinga J, Gerstenbluth I, Duits A, Tami A, Bailey A. Understanding risk communication for prevention and control of vector-borne diseases: A mixed-method study in Curacao. PLoS Negl Trop Dis. 2020;14:e0008136. 10.1371/journal.pntd.0008136 PMC7153856 32282848

[4] Kotsakiozi P, Gloria-Soria A, Caccone A, Evans B, Schama R, Martins AJ (2017). Tracking the return of *Aedes aegypti* to Brazil, the major vector of the dengue, chikungunya and Zika viruses. PLoS Negl Trop Dis.

[5] Zellweger RM, Cano J, Mangeas M, Taglioni F, Mercier A, Despinoy M (2017). Socioeconomic and environmental determinants of dengue transmission in an urban setting: an ecological study in Noumea, New Caledonia. PLoS Negl Trop Dis.

[6] Bulthuis SE, Kok MC, Raven J, Dieleman MA (2019). Factors influencing the scale-up of public health interventions in low- and middle-income countries: a qualitative systematic literature review. Health Policy Plan.

[7] WHO (2007). Everybody business: strengthening health systems to improve health outcomes: WHO’s framework for action.

[8] WHO (2010). Monitoring the building blocks of health systems: a handbook of indicators and their measurement strategies.

[9] Sacks E, Morrow M, Story WT, Shelley KD, Shanklin D, Rahimtoola M (2018). Beyond the building blocks: integrating community roles into health systems frameworks to achieve health for all. BMJ Glob Health.

[10] Mounier-Jack S, Griffiths UK, Closser S, Burchett H, Marchal B (2014). Measuring the health systems impact of disease control programmes: a critical reflection on the WHO building blocks framework. BMC Public Health.

[11] Manyazewal T (2017). Using the World Health Organization health system building blocks through survey of healthcare professionals to determine the performance of public healthcare facilities. Arch Public Health.

[12] Obermann K, Chanturidze T, Richardson E, Tanirbergenov S, Shoranov M, Nurgozhaev A (2016). Data for development in health: a case study and monitoring framework from Kazakhstan. BMJ Glob Health.

[13] Mutale W, Bond V, Mwanamwenge MT, Mlewa S, Balabanova D, Spicer N (2013). Systems thinking in practice: the current status of the six WHO building blocks for health system strengthening in three BHOMA intervention districts of Zambia: a baseline qualitative study. BMC Health Serv Res.

[14] Yu D, Souteyrand Y, Banda MA, Kaufman J, Perriens JH (2008). Investment in HIV/AIDS programs: does it help strengthen health systems in developing countries?. J Glob Health.

[15] Abejirinde IO, Ingabire CM, van Vugt M, Mutesa L, van den Borne B, Busari JO (2018). Qualitative analysis of the health system effects of a community-based malaria elimination program in Rwanda. Res Rep Trop Med.

[16] CBS: General facts Curaçao. https://www.cbs.cw/website/general-facts-curacao_3169/ (2019). Accessed 19 Feb 2020.

[17] Downe-Wamboldt B (1992). Content analysis: method, applications, and issues. Healthc Women Int.

[18] Nonaka D, Jimba M, Mizoue T, Kobayashi J, Yasuoka J, Ayi I (2012). Content analysis of primary and secondary school textbooks regarding malaria control: a multi-country study. PLoS ONE..

[19] Bastani P, Samadbeik M, Dinarvand R, Kashefian-Naeeini S, Vatankhah S (2018). Qualitative analysis of national documents on health care services and pharmaceuticals` purchasing challenges: evidence from Iran. BMC Health Serv Res.

[20] Bowen GA (2009). Document analysis as a qualitative research method. Qual Res J.

[21] Greaves DE (2016). Health management/leadership of small Island developing states of the English-speaking Caribbean: a critical review. J Health Manag.

[22] Doyle J, Asante A, Roberts G. Human resources for health (HRH) issues and challenges in 13 Pacific Islands countries. Australia; 2011.

[23] Scandurra G, Romano AA, Ronghi M, Carfora A (2018). On the vulnerability of small Island developing states: a dynamic analysis. Ecol Ind.

[24] Senkubuge F, Modisenyane M, Bishaw T (2014). Strengthening health systems by health sector reforms. Glob Health Action.

[25] Cooke RA, Szumal JL (1993). Measuring normative beliefs and shared behavioral expectations in organizations: the reliability and validity of the organizational culture inventory. Psychol Rep.

[26] Cooke RA, Szumal JL (1994). The impact of group interaction styles on problem-solving effectiveness. J Appl Behav.

[27] Sande S, Zimba M, Nyasvisvo D, Mukuzunga M, Kooma EH, Mberikunashe J (2019). Getting ready for integrated vector management for improved disease prevention in Zimbabwe: a focus on key policy issues to consider. Malar J.

[28] Chanda E, Govere JM, Macdonald MB, Lako RL, Haque U, Baba SP (2013). Integrated vector management: a critical strategy for combating vector-borne diseases in South Sudan. Malar J.

[29] Herdiana H, Sari JFK, Whittaker M (2018). Intersectoral collaboration for the prevention and control of vector borne diseases to support the implementation of a global strategy: a systematic review. PLoS ONE..

[30] WHO (2012). Monitoring and evaluation indicators for integrated vector management.

