# Supplementary material for: Evaluating and strengthening the health system of Curaҫao to improve its performance for future outbreaks of vector-borne diseases
Source: Parasit Vectors. 2021 Sep 26;14:500. doi: 10.1186/s13071-021-05011-x (PMC8474927; doi:10.1186/s13071-021-05011-x)
Supplement: Supplementary file 1 — Additional file 1: Table S1. Characteristics of the study participants [file 13071_2021_5011_MOESM1_ESM.docx]

**Table S1.** Characteristics of the study participants

| **Focus Group Discussion with Professionals (*n =* 30)** | | | | |
| --- | --- | --- | --- | --- |
|  | **Number of participants** | **Age range** | **Gender**^a^ | **Expertise (s)** |
| 1 | 9 | 25-61 | 5F/4M | Vector control, surveillance of vector and breeding sites |
| 2 | 6 | 26-40 | 2F/4M | Vector control, surveillance of vector and breeding sites |
| 3 | 3 | 47-70 | 2F/1M | Previous ministers of health |
| 4 | 5 | 57-64 | 1F/4M | Epidemiology, vector control, policy, surveillance of cases, vector and breeding sites, microbiology, laboratory techniques, and procedures, entomology, microbiology |
| 5 | 7 | 36-63 | 4F/3M | Vector control, surveillance of vector and breeding sites |
| **Expert Interviews (*n =* 11)** | | | | |
|  | **Name**^c^ | **Age** | **Gender** | **Profession (s)** |
| 1 | Sarah | 59 | F | Epidemiologist |
| 2 | John | 59 | M | Previous head of the sector of health/ epidemiologist/ general practitioner |
| 3 | Elsa | 52 | F | Entomologist/ policymaker/ registered restricted pest controler |
| 4 | Ana | 63 | F | Coordinator of the VCU^b^ |
| 5 | Peter | 62 | M | Coordinator of the VCU |
| 6 | Stephan | 57 | M | Policy-maker/ general practitioner/ previous director of the sector of health |
| 7 | Glen | 66 | M | Previous minister of health/ general practitioner |
| 8 | Sol | 69 | F | Geriatrician |
| 9 | Audrey | NA | F | Laboratory technician |
| 10 | Twin | 81 | F | Alternative medicine practitioner |
| 11 | Sandro | 66 | M | General practitioner |

Additional notes:

-The majority of the interviewed health professionals worked for the MoHEN during the period of data collection of this study. Except for the following participants; (i) previous ministers of health who worked from 10-10-2010 to 29-09-2012 (DD-MM-YYYY), (Glen) 31-12-2012 to 30-11-2015, 30-11-2015 to 23-12-2016, (ii) a previous deputy who worked in 2002, (iii) the geriatrician who worked from 2007 to 2015, and (iv) the microbiologist who worked from 2008 to 2016.

- The first FGD with health professionals was held in January 2019, and the last one took place in October 2019.

- The first interview with an expert was held in January 2019, and the last one took place in December 2020.

^a^ Gender: F= Female, and M=Male.

^b^VCU=Vector Control Unit

^c^ Fictional names were used to protect the identity of the study participants.
