# Supplementary material for: Evaluating and strengthening the health system of Curaҫao to improve its performance for future outbreaks of vector-borne diseases
Source: Parasit Vectors. 2021 Sep 26;14:500. doi: 10.1186/s13071-021-05011-x (PMC8474927; doi:10.1186/s13071-021-05011-x)
Supplement: Supplementary file 2 — Additional file 2: Table S2. Characteristics of the collected documents [file 13071_2021_5011_MOESM2_ESM.docx]

**Table S2.** Characteristics of the collected documents

|  | **Type of document** | **Amount** | **Department (s)** |
| --- | --- | --- | --- |
| 1 | Legislation, policy, protocols | 4 | Policy Department |
| 2 | Plans (e.g., project, business, surveillance, communication plan) | 13 | Director of the health sector, Policy Department, ERU |
| 3 | Health promotion materials | 14 | Department of communication |
| 4 | Evaluation reports | 4 | External medical entomologists, Court of Audit Curaҫao |
| 5 | Minutes of meetings | 5 | Director of the health sector, Policy Department, ERU, VCU |
| 6 | Budget of MoHEN | 2 | Policy Department |
| 7 | Surveillance reports | 8 | ERU, VCU |
|  | **Total** | **50** |  |
