# Supplementary material for: Evaluating and strengthening the health system of Curaҫao to improve its performance for future outbreaks of vector-borne diseases
Source: Parasit Vectors. 2021 Sep 26;14:500. doi: 10.1186/s13071-021-05011-x (PMC8474927; doi:10.1186/s13071-021-05011-x)
Supplement: Supplementary file 3 — Additional file 3: Text S1. Topic guide: FGD with the Vector Control Unit [file 13071_2021_5011_MOESM3_ESM.docx]

**Text S1.** Topic guide: FGD with the Vector Control Unit

**Topic guide: Understanding the preparedness and performance of the health system, and risk communication in the face of chikungunya and Zika virus infection epidemics**

**Group:** Vector control inspectors

**FGD number : ……………………………… Moderator : ………………………………**

**Date : ……………………………… Note-taker : ………………………………**

**Introduce yourself to the participants:**

Thank you very much for agreeing to participate in this group discussion. My name is Vaitiare Jansen. I am a doctoral student at the University of Groningen.

- ***Explain the general purpose of the study***: The general purpose of the study is to understand risk communication and the performance of the health system from your point of view in order to provide the health system with content specific advice to strengthen risk communication efforts and sustainability of risk management.
- ***Estimated time***: Approximately 1 ½ hour
- ***Right to participate and withdraw from the study:*** Involvement in this study is entirely voluntary. You are free to withdraw from the study at any time. You are free to skip any questions that you would prefer not to answer during the discussion.
- ***Use of tape recorder***: To be able to keep a more accurate record of our discussion, I am proposing to use a tape recorder, if you do not mind. Do you mind if I use a tape recorder? *(observe whether people agrees)*
- ***Plan to protect the identity of the participants:*** The information that we will discuss here today will remain anonymous. Your names will be removed from the data, and no one will be able to link your name with what is said. No one apart from the research team will have access to the data. This data will be published and shared with the scientific community, but your name will not appear in any of the publications.
- ***Basic principles:***

1. Respecting opinions from others is important.
2. There are no right and wrong answers. We value each idea, opinion and experience.
3. One person speaks at a time.
4. Ask if there is any question.

- Do you have any questions?
- ***Consent:*** Do you agree to take part in this discussion?
- The moderator turns on the digital recorder and starts the discussion

**Introduction**

- As an introduction, let us go around so that you can introduce yourselves and tell us your name, age and what type of work you do.

**Let us start our discussion by talking about chikungunya and Zika. Curaçao has witnessed the chikungunya virus infection outbreak in 2014-2015 and, more recently, the Zika virus infection outbreak in 2016. As inspectors of the Vector Control Unit of the GMN, you worked closely with the community in order to reduce cases of chikungunya and Zika virus infection.**

1. ***What do you think about the job of a vector control inspector?***

**Probe for:**

1. What are the good things?
2. What can be better?
3. Do you think that the job of a vector control inspector is important?
4. Why?

**Topic 1: Preparedness of the health system**

1. ***What does preparing for an outbreak of diseases transmitted by mosquitoes mean to you?***
2. ***How prepared was the health system for chikungunya?***

**Probe for:**

1. In what way did they prepared?
2. What about the vector control unit?
3. Ask for the following factors *(enough workforce and materials)* if they are not mentioned.
4. ***How prepared was the health system for Zika?***

**Probe for:**

1. In what way did they prepared?
2. What about the vector control unit?
3. Ask for the following factors *(enough workforce and materials)* if they are not mentioned.
4. ***What can be done to improve the readiness of the health system?***

**Probe for:**

1. Why?
2. What can be done to improve the readiness of the vector control unit?

**Topic 2: Performance of the health system**

1. ***What is the role of the vector control unit in the health system?***

**Probe for:**

1. Which departments work closely with the department of vector control?
2. How is the collaboration between the departments?
3. ***Could you describe your experiences during the chikungunya epidemic as vector control inspectors?***

**Probe for:**

1. What went well?
2. What can be improved?
3. ***Could you describe your experiences during the Zika epidemic as vector control inspectors?***

**Probe for:**

1. What went well?
2. What can be improved?

**We heard in the community that many people thought that the government did anything.**

1. ***Why do people think like this?***

**Probe for:**

1. Which challenges do you face during your work?
2. Ask for the following *factors (trust issues, limited resources, immigrants, unplanned urbanisation, and work-related insecurities)* if they are not mentioned.

**Now that we spoke about the challenges that you faced or that you are facing during your work.**

1. ***I would like to know how do these challenges affect your day-to-day work?***
2. ***What lessons did the vector control unit learn from these epidemics?***

**Probe for:**

1. What has been done with the lessons learned?
2. ***What could be done to improve the performance of the vector control unit?***

**Probe for:**

1. Why?
2. Who can provide the vector control unit with the needed help?

**Topic 3: Risk communication**

**We heard that the vector control inspectors were also responsible for the provision of information to the community.**

1. ***What type of information was or is shared with the community?***

**Probe for:**

1. Ask for the following type of information *(preventive measures, the transmission of disease, and treatment)* if they are not mentioned.
2. ***Do you think that the information you provided helped to change the behaviour of people?***
3. ***Which challenges do vector control inspectors face when they are providing information to the community?***

**Probe for:**

1. Ask for the following factors *(language barriers, lack of self-confidence, information materials, time, and community interest)* if they are not mentioned.
2. ***What can be done to improve communication between the vector control inspectors and the community?***

**Closing question**

1. ***Imagine, this year, we have another disease transmitted by mosquitoes. Do you think we are prepared to deal with it?***

**Probe for:**

1. What can be done?

We are now reaching the end of the discussion. Does anyone have any further comments to add before we conclude this group discussion? I want to thank you all very much for your participation in this discussion; your experiences and opinions are valuable to assist in improving risk communication and risk management in Curaçao.
