# Supplementary material for: Evaluating and strengthening the health system of Curaҫao to improve its performance for future outbreaks of vector-borne diseases
Source: Parasit Vectors. 2021 Sep 26;14:500. doi: 10.1186/s13071-021-05011-x (PMC8474927; doi:10.1186/s13071-021-05011-x)
Supplement: Supplementary file 4 — Additional file 4: Text S2. Topic guide FGD with health professionals [file 13071_2021_5011_MOESM4_ESM.docx]

**Text S2.** Topic guide FGD with health professionals

**Topic guide: Understanding the preparedness and performance of the health system, and risk communication in the face of dengue, chikungunya and Zika virus infection epidemics**

**Group:** Health professionals

**FGD number : ……………………………… Moderator : ………………………………**

**Date : ……………………………… Note-taker : ………………………………**

**Introduce yourself to the participants:**

Thank you very much for agreeing to participate in this group discussion. My name is Vaitiare Jansen. I am a doctoral student at the University of Groningen.

- ***Explain the general purpose of the study***: The general purpose of the study is to understand risk communication and the performance of the health system from your point of view, in order to provide the health system with content specific advice to strengthen risk communication efforts and sustainability of risk management.
- ***Estimated time***: Approximately 1 ½ hour
- ***Right to participate and withdraw from the study:*** Involvement in this study is entirely voluntary. You are free to withdraw from the study at any time. You are free to skip any questions that you would prefer not to answer during the discussion.
- ***Use of tape recorder***: To be able to keep a more accurate record of our discussion, I am proposing to use a tape recorder, if you do not mind. Do you mind if I use a tape recorder? *(observe whether people agrees)*
- ***Plan to protect the identity of the participants:*** The information that we will discuss here today will remain anonymous. Your names will be removed from the data, and no one will be able to link your name with what is said. No one apart from the research team will have access to the data. This data will be published and shared with the scientific community, but your name will not appear in any of the publications.
- ***Basic principles:***

1. Respecting opinions from others is important.
2. There are no right and wrong answers. We value each idea, opinion and experience.
3. One person speaks at a time.
4. Ask if there is any question.

- Do you have any questions?
- ***Consent:*** Do you agree to take part in this discussion?
- The moderator turns on the digital recorder and starts the discussion

**Introduction**

- As an introduction, let us go around so that you can introduce yourselves and tell us your name, age and what type of work you do.

**Let us start our discussion by talking about chikungunya and Zika. Curaçao has witnessed the chikungunya virus infection outbreak in 2014-2015 and, more recently, the Zika virus infection outbreak in 2016. Dengue epidemics occur cyclically. As health professionals of the GMN, you worked closely with the community to reduce the risk for dengue, chikungunya and Zika virus infection.**

1. ***What does preparing for an outbreak of diseases transmitted by mosquitoes mean to you?***
2. ***How prepared was the health system for dengue?***

**Probe for:**

1. How prepared was the health system for chikungunya?
2. Which department (s) were less prepared, and why?
3. How prepared was the health system for Zika?
4. ***What can be done to improve the readiness of the health system?***

**Probe for:**

1. Why?

**Topic 1: Governance**

1. ***How was the health system organised during the epidemic of dengue in 2010?***

***Probe for:***

1. Which departments were responsible for the surveillance of cases, vector, communication, prevention and control?
2. Which ministries/departments/institutions were involved and needed to work together?
3. ***How was the health system organised during the epidemic of chikungunya in 2014-2015?***

**Probe for:**

1. What were the changes *(e.g., assigned new head of department, laws, protocols, strategies for prevention and control of the vector?)*
2. How was the health system organised during the epidemic of Zika in 2016?
3. What were the changes?
4. Did the collaboration between ministries/departments/institutions improve or deteriorate in the last ten years?
5. Currently, how is the collaboration between departments?
6. What do you think about the organisational structure of the ministry of health (MoH)?
7. ***What were the challenges of the MoH during the epidemics of dengue and chikungunya?***

**Probe for:**

1. What were the challenges with regards to the development, implementation and reinforcement of laws/protocols?
2. What were the challenges with regards to the surveillance of cases and the vector?
3. What were the challenges concerning the prevention and control of the vector?
4. What were the challenges concerning risk communication?
5. ***What were the challenges of the MoH during the epidemic of Zika?***
6. ***Which laws or protocols did you use for public health issues, surveillance of cases, surveillance of vector, prevention and control of the vector?***

**Probe for:**

1. There is a basic law on infection disease *(Bestrijding van besmettelijk ziekten, p.b.1921, no.66).* Is this law still useful?
2. What are the benefits and disadvantages of this law or the protocols that you used?
3. What about laws concerning public health and prenatal Zika infection?
4. ***What are the lessons learned?***

**Probe for:**

1. What have you done with these lessons?
2. What is needed to improve surveillance of cases, vector, prevention and vector control?

**Topic 2: Financing system**

1. ***Describe the healthcare financing system?***

**Probe for:**

1. Which stakeholders *(e.g., hospital, general practitioners, specialists, laboratories)* are involved?
2. What are the benefits and gaps in this healthcare financing system?
3. ***How was the public health sector financing system organised during the dengue epidemic?***

**Probe for:**

1. Which ministries/departments/institutions were involved?
2. What are the benefits and gaps in this public health financing system?
3. What were the major changes during the chikungunya and Zika epidemics in the context of financing?
4. ***Which services related to prevention and vector control are free for the community?***

**Probe for:**

1. Which services are not free?
2. Why?
3. ***What can be done to improve the public health sector financing system to ensure prevention and vector control sustainability?***

**Topic 3: Health information system**

1. ***What type of data (e.g., cases of VBDs, the vector etc.) has been collected by which department in the last ten years?***

**Probe for*:***

1. How was the data collected?
2. How was the data stored?
3. What was the aim of the data collection?
4. How reliable was the collected data?
5. ***How was the data analysed?***

**Probe for:**

1. By whom?
2. What were the strengths and gaps in the collected data?
3. ***Has the data been published or shared?***

**Probe for:**

1. By whom?
2. To whom *(e.g., community, public health system)?*
3. Was the information shared on time?
4. What are the strengths and gaps in data sharing?
5. ***How was the risk communication regarding VBDs organised?***

**Probe for:**

1. How was the risk communication regarding dengue communicated to the community?
2. How was the risk communication regarding chikungunya and Zika communicated to the community?
3. How was the risk communication regarding VBDs communicated within the health system?
4. What were the strengths and gaps in risk communication strategies?
5. What can be done to improve risk communication?
6. ***How was the surveillance system of cases of VBDs organised?***

**Probe for:**

1. Which surveillance methods, software and strategies were used during the dengue, chikungunya and Zika epidemics?
2. Which department was responsible for the surveillance of cases?
3. Which departments and institutions needed to collaborate?
4. How was the collaboration?
5. Was the collaboration documented in law or protocol?
6. What can be done to improve the surveillance system of cases?
7. ***How was the surveillance system of the vector organised?***

***Probe for:***

1. Which surveillance methods, software and strategies were used during the dengue, chikungunya and Zika epidemics?
2. Which department was responsible for the surveillance of the vector?
3. Which departments and institutions needed to collaborate?
4. How was the collaboration?
5. Was the collaboration documented in law or protocol?
6. What can be done to improve the surveillance system of the vector?

**Topic 4: Workforce**

1. ***How qualified are the workforce of the health system? Explain why?***

**Probe for:**

1. Is it enough?
2. Are the job descriptions clear?
3. What are the strengths and gaps in the workforce of the VCU?
4. What are the strengths and gaps in the workforce of the department of epidemiology and research?
5. What are the strengths and gaps in the workforce of the department of communication?
6. What are the strengths and gaps in the workforce of the policy department?
7. ***How satisfied were you with your job during the epidemics?***

**Probe for:**

1. Were you motivated?
2. Which factors were associated with a reduction in motivation and satisfaction?
3. ***Which stakeholders (e.g., general practitioners, hospital, laboratories, department of communication, epidemiology and research, THZ, Selikor, DOW, PR, Policy) are essential for prevention and vector control?***

**Probe for:**

1. *Why?*
2. ***What can be done to improve the performance of the workforce?***

**Topic 5: Service delivery**

1. ***Which interventions were conducted by the public health system during the epidemic of dengue?***

**Probe for:**

1. Which interventions (related or not related to the community) were conducted during the epidemics of chikungunya and Zika, and by whom?
2. What are the strengths and gaps in these interventions?
3. What can be done to improve these interventions?
4. ***What can be done to ensure the sustainability of these interventions?***
5. ***Which resources were needed to perform these interventions?***

**Probe for:**

1. Which resources were used for prevention and vector control?
2. Which biological methods for vector control (all stages) were used?
3. Which chemical methods for vector control were used?
4. Which other methods were used?
5. Were the resources available during the epidemics? Why?
6. Which measures were taken to protect the workforce during fieldwork?
7. Are these interventions documented?
8. **How effective were the performed interventions?**

**Probe for:**

1. Were the performed interventions sufficient? Why?
2. Have the interventions been evaluated?
3. Why?

**Topic 6: Medical products and technology**

1. ***Which laboratories worked with the MoH during the epidemics of dengue, chikungunya and Zika?***

**Probe for:**

1. What were their tasks?
2. What were the challenges?
3. How was the collaboration?
4. Was this collaboration documented in a protocol?
5. ***Which diagnostic tests were performed to test for dengue, chikungunya and Zika?***

**Probe for:**

1. Other diseases transmitted by mosquitos were also tested?
2. Did the laboratories take “cross-reactivity” also into account?
3. Did the laboratories perform a “confirmatory test *(virus neutralisation test)”?*
4. Is a protocol concerning testing for VBDs for the general practitioners available?
5. Explain the content of this protocol?
6. What are the strengths and gaps of this protocol?
7. ***What were the challenges concerning access to medication to reduce the symptoms of mentioned VBDs?***

**Closing question**

1. ***Imagine, this year, we have another disease transmitted by mosquitoes. Do you think we are prepared to deal with it?***

**Probe for:**

1. What can be done?

We are now reaching the end of the discussion. Does anyone have any further comments to add before we conclude this group discussion? I want to thank you all very much for your participation in this discussion; your experiences and opinions are valuable to assist in improving risk communication and risk management in Curaçao.
