# Supplementary material for: Evaluating and strengthening the health system of Curaҫao to improve its performance for future outbreaks of vector-borne diseases
Source: Parasit Vectors. 2021 Sep 26;14:500. doi: 10.1186/s13071-021-05011-x (PMC8474927; doi:10.1186/s13071-021-05011-x)
Supplement: Supplementary file 5 — Additional file 5: Text S3. Topic guide: Interview with general practitioners and geriatrician [file 13071_2021_5011_MOESM5_ESM.docx]

**Text S3.** Topic guide: Interview with general practitioners and geriatrician

**Topic guide for interviews: Understanding the preparedness and performance of the health system, and risk communication in the face of dengue, chikungunya and Zika virus infection epidemics**

**IDI number: ………………………………**

**Date: ………………………………**

**Interviewer: ………………………………**

**Introduce yourself to the participants:** Thank you very much for agreeing to participate in this research. My name is Vaitiare Mulderij-Jansen. I am a doctoral student at the University of Groningen.

- ***Explain the general purpose of the study***: The general purpose of the study is to understand risk communication and behaviour of individuals concerning the prevention and control of chikungunya, dengue, and Zika, from your point of view. Your perceptions, opinions and experiences can help us provide the government with content specific advice to strengthen risk communication efforts, the sustainability of risk management and enhance the health-seeking behaviour of people living in Curaҫao.
- ***Estimated time***: Approximately 1 hour
- ***Right to participate and withdraw from the study:*** Involvement in this study is entirely voluntary. You are free to withdraw from the study at any time. You are free to skip any questions that you would prefer not to answer during the interview.
- ***Use of tape recorder***: To be able to keep a more accurate record of the interview, I am proposing to use a tape recorder, if you do not mind. Do you mind if I use a tape recorder? *(observe whether people agrees)*
- ***Plan to protect the identity of the participants:*** The information that we will discuss here today will remain anonymous. Your name will be removed from the data, and no one will be able to link your name with what is said. No one apart from the research team will have access to the data. This data will be published and shared with the scientific community, but your name will not appear in any of the publications.
- ***Basic principles:***

1. There are no right and wrong answers. I value each idea, opinion and experience.
2. Ask if there is any question.

- Do you have any questions?
- ***Consent:*** Sign the “informed consent” form.
- The interviewer turns on the digital recorder and starts with the interview.

**Introduction**

- As an introduction, can you introduce yourself, tell me your name, age, and whether you are currently working, and what type of work you do.

Let us start the interview by talking about chikungunya, dengue, and Zika. The majority of individuals living in Curaçao witnessed the dengue outbreak in 2010, the chikungunya outbreak in 2014-2015 and more recently, the Zika outbreak in 2016.

1. ***What do you know about dengue?***

**Probe for:**

1. Ask for the following types of information *(e.g. transmission routes, prevention measures, the link between these diseases, symptoms, treatment)* if they are not mentioned.
2. ***What do you know about chikungunya and Zika?***

**Probe for:**

1. Ask for the following types of information *(e.g. transmission routes, prevention measures, the link between these diseases, symptoms, treatment)* if they are not mentioned.
2. ***According to you, what are the reasons/causes of these outbreaks in Curaçao?***

**Probe for:**

1. What makes Curaçao susceptible to these diseases?
2. ***How did you obtain or receive information about these diseases?***

**Topic 1: Preparedness**

1. ***How prepared was the health system for dengue?***

**Probe for:**

- 1. How prepared was the health system for chikungunya and Zika?
  2. Did the level of preparedness of the health system influence the preparedness of the general practitioners?
  3. Explain how?

1. ***What can be done to improve the preparedness of the health system?***

**Topic 2: Performance of the health system**

1. ***What was your role during the epidemics of dengue, chikungunya and Zika?***

**Probe for:**

- 1. What were your challenges during the dengue epidemic *(e.g., communication problems with patients or other general practitioners, lack of information, protocol)*?
  2. What were your challenges during the epidemic of chikungunya?
  3. And Zika?

1. ***How was the collaboration between general practitioners and the MoH during these epidemics?***

**Probe for:**

- 1. What went well, and what can be improved?
  2. What can be done to improve the collaboration between the MoH and the general practitioners?

1. ***How was the collaboration between general practitioners and laboratories during these epidemics?***

**Probe for:**

- 1. What went well, and what can be improved?
  2. What can be done to improve the collaboration between general practitioners and laboratories?

**Topic 3: Communication**

1. ***How was the communication concerning dengue between general practitioners and the association of general practitioners?***

**Probe for:**

- 1. Did you receive the information on time?
  2. Was the information sufficient and clear?
  3. Which type of information did you miss?

1. ***How was the communication concerning chikungunya and Zika between general practitioners and the association of general practitioners?***

**Probe for:**

a. Did you receive information on time?

b. Was the information sufficient and clear?

c. Which type of information did you miss?

1. ***What can be done to improve the communication between general practitioners and the association of general practitioners?***

**Probe for:**

- 1. How do you want to receive information?
  2. When do you want to receive information?
  3. Which type of information do you want to receive?

1. ***What do you think about the communication strategies performed by the MoH during the last three epidemics?***

**Probe for:**

- 1. What went well, and what can be improved?
  2. Was the communication strategies effective and efficient? Why?
  3. What can be done to improve the communication strategies of the MoH?

**Topic 4: Prevention and control of VBDs**

1. ***Who is responsible for prevention and vector control?***

**Probe for:**

1. Ask for the following individuals/groups/institutions *(e.g., community, government)* if they are not mentioned.
2. Why?
3. ***According to you, which factor did obstruct the prevention and control strategies of the Moh?***

**Probe for:**

1. Ask for the following factors *(e.g., financing, lack of workforce, materials, law, the collaboration between stakeholders)* if they are not mentioned.
2. What can be done to improve the health-seeking behaviour of the community?
3. What can be done to improve the collaboration between the MoH and the community?

**Closing question**

1. ***Imagine, this year, we have another disease transmitted by mosquitoes. Do you think we are prepared to deal with it?***

**Probe for:**

1. What can be done?

We are now reaching the end of the interview. Do you have any further comments to add before we conclude? Thank you very much for your participation in this interview; your experiences and opinions are valuable to assist in improving risk communication and risk management in Curaçao.
