# Supplementary material for: Evaluating and strengthening the health system of Curaҫao to improve its performance for future outbreaks of vector-borne diseases
Source: Parasit Vectors. 2021 Sep 26;14:500. doi: 10.1186/s13071-021-05011-x (PMC8474927; doi:10.1186/s13071-021-05011-x)
Supplement: Supplementary file 7 — Additional file 7: Text S5. Topic guide: Interview with a laboratory technician [file 13071_2021_5011_MOESM7_ESM.docx]

**Text S5.** Topic guide: Interview with a laboratory technician

**Topic guide for interviews: Understanding the preparedness and performance of the health system in the face of dengue, chikungunya and Zika virus infection epidemics**

**IDI number: ………………………………**

**Date: ………………………………**

**Interviewer: ………………………………**

**Introduce yourself to the participants:** Thank you very much for agreeing to participate in this research. My name is Vaitiare Mulderij-Jansen. I am a doctoral student at the University of Groningen.

- ***Explain the general purpose of the study***: The general purpose of the study is to understand the risk communication and behaviour of individuals concerning the prevention and control of chikungunya, dengue, and Zika, from your point of view. Your perceptions, opinions and experiences can help us provide the government with content specific advice to strengthen risk communication efforts, the sustainability of risk management and enhance the health-seeking behaviour of people living in Curaҫao.
- ***Estimated time***: Approximately 1 hour
- ***Right to participate and withdraw from the study:*** Involvement in this study is entirely voluntary. You are free to withdraw from the study at any time. You are free to skip any questions that you would prefer not to answer during the interview.
- ***Use of tape recorder***: To be able to keep a more accurate record of the interview, I am proposing to use a tape recorder, if you do not mind. Do you mind if I use a tape recorder? *(observe whether people agrees)*
- ***Plan to protect the identity of the participants:*** The information that we will discuss here today will remain anonymous. Your name will be removed from the data, and no one will be able to link your name with what is said. No one apart from the research team will have access to the data. This data will be published and shared with the scientific community, but your name will not appear in any of the publications.
- ***Basic principles:***

1. There are no right and wrong answers. I value each idea, opinion and experience.
2. Ask if there is any question.

- Do you have any questions?
- ***Consent:*** Sign the “informed consent” form.
- The interviewer turns on the digital recorder and starts with the interview.

**Introduction**

- As an introduction, can you introduce yourself, tell me your name, age, and whether you are currently working, and what type of work you do.

Let us start the interview by talking about chikungunya, dengue, and Zika. The majority of individuals living in Curaçao witnessed the dengue outbreak in 2010, the chikungunya outbreak in 2014-2015 and more recently, the Zika outbreak in 2016.

1. ***What do you know about dengue?***

**Probe for:**

1. Ask for the following types of information *(e.g. transmission routes, prevention measures, the link between these diseases, symptoms, treatment)* if they are not mentioned.
2. ***What do you know about chikungunya and Zika?***

**Probe for:**

1. Ask for the following types of information *(e.g. transmission routes, prevention measures, the link between these diseases, symptoms, treatment)* if they are not mentioned.
2. ***According to you, what are the reasons/causes of these outbreaks in Curaçao?***

**Probe for:**

1. What makes Curaçao susceptible to these diseases?
2. ***How did you obtain or receive information about these diseases***

**Topic 1: Preparedness and Performance**

1. ***What was the role of ADC during the epidemics of dengue, chikungunya and Zika?***

**Probe for:**

- 1. With which departments/institutions did ADC collaborate?
  2. What do you think about the collaboration between the MoH and ADC?
  3. What are the agreements between ADC and MoH?
  4. Are the agreements between ADC and the MoH documented?
  5. What can be done to improve the collaboration between these stakeholders?

1. ***How prepared was ADC for the chikungunya epidemic?***

***Probe for:***

- 1. What about the epidemic of Zika and dengue?
  2. What went well, and what can be improved?

1. ***According to you, was the MoH prepared for the chikungunya and Zika epidemics?***

**Probe for:**

- 1. Why?
  2. What went well, and what can be improved?
  3. What about the dengue epidemics?

1. ***Which type of test were conducted to test for dengue, chikungunya and Zika?***

**Probe for:**

- 1. Did the ADC test for other diseases transmitted by mosquitoes?
  2. Did the ADC take cross-reactivity between dengue and Zika into account?
  3. Did the ADC perform a confirmatory test (virus neutralisation test)?
  4. What can you tell me about the sensitivity and specificity of the tests?

1. ***How was the financing system of the tests related to public health issues organised?***

**Probe for:**

- 1. What are the strengths and gaps in this financing system?

1. ***Which measures did ADC take to ensure the quality of the tests?***
2. ***Do you work according to a protocol?***

**Probe for:**

- 1. What is the content of the protocol?

1. ***What were the challenges that ADC faced during the last three epidemics of VBDs?***
2. ***What can be done to improve the preparedness of the ADC in the context of VBDs?***

**Probe for:**

- 1. What can be done to improve the performance of the ADC?

**Topic 3: Communication**

1. ***What do you think about the communication between the Moh and ADC?***

**Probe for:**

- 1. What do you think about the communication within ADC?
  2. What are the gaps in the communication strategy?
  3. What can be done to improve the communication between the MoH and the ADC?
  4. What do you want to know about VBDs?
  5. When do you want to receive information about VBDs?
  6. How do you want to receive information about VBDs?

**Closing question**

1. ***Imagine, this year, we have another disease transmitted by mosquitoes. Do you think that ADC is prepared to deal with it?***

**Probe for:**

1. What can be done?

We are now reaching the end of the interview. Do you have any further comments to add before we conclude? Thank you very much for your participation in this interview; your experiences and opinions are valuable to assist in improving risk communication and risk management in Curaçao.
