# Supplementary material for: Evaluating and strengthening the health system of Curaҫao to improve its performance for future outbreaks of vector-borne diseases
Source: Parasit Vectors. 2021 Sep 26;14:500. doi: 10.1186/s13071-021-05011-x (PMC8474927; doi:10.1186/s13071-021-05011-x)
Supplement: Supplementary file 8 — Additional file 8: Text S6. Topic guide: FGD with previous ministers of health [file 13071_2021_5011_MOESM8_ESM.docx]

**Text S6.** Topic guide: FGD with previous ministers of health

**Topic guide: Understanding the preparedness and performance of the health system, and risk communication in the face of chikungunya and Zika virus infection epidemics**

**Group:** previous ministers of health

**FGD number : ……………………………… Moderator : ………………………………**

**Date : ……………………………… Note-taker : ………………………………**

**Introduce yourself to the participants:**

Thank you very much for agreeing to participate in this group discussion. My name is Vaitiare Jansen. I am a doctoral student at the University of Groningen.

- ***Explain the study's general purpose***: I did various group discussions, interviews with locals, specific groups in our community, and health professionals at different levels to gather information about risk communication, the preparedness, and performance of the health system of Curaҫao. Now it's your turn, as previous ministers of health, to tell me your experience with each of these epidemics. The general purpose of the study is to understand risk communication, the preparedness, and performance of the health system from your point of view in order to provide the health system with content specific advice to strengthen risk communication efforts and sustainability of risk management
- ***Estimated time***: Approximately 1 ½ hour
- ***Right to participate and withdraw from the study:*** Involvement in this study is entirely voluntary. You are free to withdraw from the study at any time. You are free to skip any questions that you would prefer not to answer during the discussion.
- ***Use of tape recorder***: To be able to keep a more accurate record of our discussion, I am proposing to use a tape recorder, if you do not mind. Do you mind if I use a tape recorder? *(observe whether people agrees)*
- ***Plan to protect the identity of the participants:*** The information that we will discuss here today will remain anonymous. Your names will be removed from the data, and no one will be able to link your name with what is said. No one apart from the research team will have access to the data. This data will be published and shared with the scientific community, but your name will not appear in any of the publications.
- ***Basic principles:***

1. Respecting opinions from others is important.
2. There are no right and wrong answers. We value each idea, opinion and experience.
3. One person speaks at a time.
4. Ask if there is any question.

- Do you have any questions?
- ***Consent:*** Do you agree to take part in this discussion?
- The moderator turns on the digital recorder and starts the discussion

**Introduction**

- As an introduction, let us go around so that you can introduce yourselves and tell us your name, age and what type of work you do.

**Let us start our discussion by talking about dengue, chikungunya and Zika. Curaçao witnessed the dengue virus infection outbreak in 2010, the chikungunya virus infection outbreak in 2014-2015 and more recently, the Zika virus infection outbreak in 2016. As previous health ministers, you worked closely with the ministry of health (MoH) to reduce the mentioned diseases' risk.**

1. ***What do you know about dengue?***

**Probe for:**

1. What do you know about chikungunya and Zika?
2. From which channels of information did you receive information about these diseases?
3. ***As minister of health, how important was the prevention and control of these diseases for you and the MoH?***

**Probe for:**

1. What was your role?
2. What actions did you perform to prevent and control these diseases?
3. What challenges did you face?
4. How important are the diseases for the current MoH?

**Topic 1: Preparedness**

1. ***What does preparing for an outbreak of diseases transmitted by mosquitoes mean to you?***
2. ***How prepared was the health system for dengue?***

**Probe for:**

1. How prepared was the health system for chikungunya?
2. And Zika?
3. What went well, and what needs to be improved?
4. ***What can be done to improve the readiness of the health system?***

**Probe for:**

1. Why?

**Topic 2: Governance**

1. ***How was the health system organised during the epidemic of dengue in 2010?***

***Probe for:***

1. Which departments were responsible for the prevention and control of this disease?
2. Which ministries/departments/institutions were involved and needed to work together?
3. How was your collaboration with these ministries/departments/institutions?
4. What went well, and what needs to be improved?
5. ***How was the health system organised during the epidemic of chikungunya in 2014-2015?***

**Probe for:**

1. What were the changes *(e.g., assigned new head of department, laws, protocols, strategies for prevention and control of the vector?)*
2. How was the health system organised during the epidemic of Zika in 2016?
3. Did the collaboration between ministries/departments/institutions improve or deteriorate in the last ten years?
4. What can you tell me about the current performance of the MoH in the context of diseases transmitted by mosquitoes?
5. Compared to your working period, what has improved and what has deteriorated?
6. Why?
7. ***What do you think about the organisational structure of the MoH?***

**Probe for:**

1. What are the strengths and gaps in the organisational structure of the MoH?
2. What can be done to improve the organisational structure of the MoH?
3. ***What were the challenges of the MoH during the epidemic of dengue?***

**Probe for:**

1. What about challenges related to communication, prevention and control strategies?
2. What were the challenges of the MoH during the epidemic of chikungunya and Zika?

I want to talk about the infrastructure of Curaҫao. Infrastructure plays an essential role in prevention and vector control. For example, there are some neighbourhoods built in a dam *(e.g., Koralspecht).* During the rainy season, these neighbourhoods have many problems with floods. Water retention leads to a higher mosquitoes density. There are more issues *(e.g., The mangroves in the city of Curaҫao)* that also impact these diseases prevention and control.

1. ***Are you aware of these problems?***
2. ***What is your role in dealing with problems related to infrastructure and health care?***

**Probe for:**

1. What are the challenges?
2. What can be done to improve the collaboration between the ministries of the government?
3. ***Which laws did you use to deal with the prevention and control of diseases transmitted by mosquitoes?***

*Tip: The basic law on infection disease (Bestrijding van besmettelijk ziekten, p.b.1921, no.66)*

**Probe for:**

1. What are the strengths and gaps in these laws?
2. The MoH made a law for public health. What is the reason that the law is not being used?
3. ***What are the lessons learned?***

**Probe for:**

1. What have you done with these lessons?

**Topic 3: Financing system**

1. ***How is the financing system of the health system organised?***

**Probe for:**

1. What do you think about this system?
2. What are the strengths and gaps in the system?
3. What can be done to improve the financing system of public health care?

**Topic 4: The sustainability of interventions**

1. ***What can be done to improve the sustainability of interventions related to diseases transmitted by mosquitoes?***

**Probe for:**

1. What is needed to improve the performance of the health system?
2. Which issue has more priority?
3. What can be done to reach this goal?
4. What can you do to help the MoH to improve its preparedness and performance to deal with future outbreaks of diseases transmitted by mosquitoes?

**Closing question**

1. ***Imagine, this year, we have another disease transmitted by mosquitoes. Do you think we are prepared to deal with it?***

**Probe for:**

1. What can be done?

We are now reaching the end of the discussion. Does anyone have any further comments to add before we conclude this group discussion? I want to thank you all very much for your participation in this discussion; your experiences and opinions are valuable to assist in improving risk communication and risk management in Curaçao.
