# Supplementary material for: Evaluating and strengthening the health system of Curaҫao to improve its performance for future outbreaks of vector-borne diseases
Source: Parasit Vectors. 2021 Sep 26;14:500. doi: 10.1186/s13071-021-05011-x (PMC8474927; doi:10.1186/s13071-021-05011-x)
Supplement: Supplementary file 9 — Additional file 9: Table S3. Coding list [file 13071_2021_5011_MOESM9_ESM.docx]

**Table S3.** Coding list

| **Code category** | **Code** | |
| --- | --- | --- |
|  | **Inductive** | **Deductive** |
| Leadership/Governance | Law  Negative image  Positive image  Priority  The visibility of the governmental departments  Power  Impact of 2010 (autonomy of Curacao)  Copy-paste the health system of the Netherlands  Consulting agency  Functional illiteracy | Governmental structure  Protocol concerning VBDs  Guidance  Collaboration  Accountability  Lawmakers  Programs  Division of tasks  Decision making |
|  |  |  |
| Financing system | Economize  Priority | Budget for prevention  Budget for care  Health insurance |
|  |  |  |
| Medical products and technologies | Collaboration  Budget  Coordination | Laboratories  Equipment |
|  |  |  |
| Health information system | Communication to the GP’s | Surveillance system cases  Surveillance system vector  Risk communication  Communication flowchart  Share information  Digitial system  Communication channels |
|  |  |  |
| Workforce | Respect  Accountability  Security during working  hours  Materials needed  Retirement  Negligence  Research team  Risk communication team  Vector control team  Promotion | Education/training  Collaboration between departments/ institution  Motivation  Guidance  Collaboration between co-workers  Job description |
|  |  |  |
| Service delivery | Vector control strategies | Availability |
| Trust | Corruption  Bureaucracy |  |
|  |  |  |
| Prevention | Budget  Prevention strategies  Impact  Future risk of other infectious diseases  Sewage system  Culex  Aedes  Cesspool | Larvacide  Bti  Personal protection against mosquitoes |
|  |  |  |
| Evaluation | Evaluation efforts |  |
| Recommendation | Collaboration  Consistency  Share information within and outside the health system  Fines  Follow up report (after the epidemic)  Enhance communication to the general practitioners  Use social platforms  Adaptation of law with regards to the infrastructure of Curaҫao | Capacity building  Governmental structure  Proactive approach  Environmental police officers  Improve waste management  Improve employee accountability |
