# Supplementary material for: Evaluating and strengthening the health system of Curaҫao to improve its performance for future outbreaks of vector-borne diseases
Source: Parasit Vectors. 2021 Sep 26;14:500. doi: 10.1186/s13071-021-05011-x (PMC8474927; doi:10.1186/s13071-021-05011-x)
Supplement: Supplementary file 10 — Additional file 10: Table S4. List of mosquito species of Curaҫao [file 13071_2021_5011_MOESM10_ESM.docx]

**Table S4. List of mosquito species of Curaҫao**

|  | **Species** | **Host** | **Status** | **Reported** | **Pathogens** |
| --- | --- | --- | --- | --- | --- |
| 1. | *Anopheles pseudopunctipennis* | Mammals | Native | Van der Kuyp, 1949 | Malaria parasites |
| 2. | *Aedes aegypti* | Human | Native | Van der Kuyp, 1949 | Dengue, Zika, chikungunya virus, Dirofilaria immitis |
| 3. | *Aedes taeniorhynchus* | Mammals, birds, reptiles | Native | Van der Kuyp, 1949 | Eastern equine encephalitis virus (EEE), Venezuelan equine encephalitis virus (VEE), Dirofilaria immitis |
| 4. | *Haemagogus chrysochlorus* | Mammals | Native | Van der Kuyp, 1949 | - |
| 5. | *Psorophora confinnis* | Mammals | Native | Van der Kuyp, 1949 | VEE virus |
| 6. | *Culex maracayensis* | - | Native | Van der Kuyp, 1949 |  |
| 7. | *Culex nigripalpus* | Mammals, birds, reptiles, batrachians | Native | Van der Kuyp, 1949 | Saint Louis encephalitis virus (SLE), EEE virus |
| 8. | *Culex quinquefasciatus* | Mammals, birds | Native | Van der Kuyp, 1949 | West Nile virus (WN), Dirofilaria immitis, Lymphatic filariasis |
| 9. | *Culex erraticus* | Mammals, birds, reptiles | Native | Van der Kuyp, 1949 | EEE, VEE virus |
| 10. | *Deinocerites magnus* | Mammals, birds, reptiles | Native | Van der Kuyp, 1949 |  |
| 11. | *Wyeomyia celaenocephala* | Mammals | Native | Van der Kuyp, 1949 |  |

Adapted from Surveillance and control of vector species in Curaҫao (West Indies): Situation analysis an needs assessment, by Francis Schaffner and Marieta Braks, 2016. Adapted with permission.
