# Supplementary material for: Evaluating and strengthening the health system of Curaҫao to improve its performance for future outbreaks of vector-borne diseases
Source: Parasit Vectors. 2021 Sep 26;14:500. doi: 10.1186/s13071-021-05011-x (PMC8474927; doi:10.1186/s13071-021-05011-x)
Supplement: Supplementary file 11 — Additional file 11: Table S5. The workforce during the epidemics compared with the required workforce to perform prevention and control strategies with regards to VBDs [file 13071_2021_5011_MOESM11_ESM.docx]

**Table S5.** The workforce during the epidemics in comparison with the required workforce to perform prevention and control strategies with regards to VBDs

| **Departments** | **The estimated workforce**  **(The business plan of the MoHEN)** | **The available workforce during the epidemics of VBDs** |
| --- | --- | --- |
| Epidemiology and Research | 1 head of the department  2 medical doctors specialised in epidemiology  1 social scientist  1 economist specialised in health care  1 statistician  1 medical expert in environmental science  2 employees to support the management of the department | 1 medical doctor/ epidemiologist *(head of the department)*  2 health professionals specialised in epidemiology and public health |
| Total FTE | 9 | 3 |
| VCU | 1 head of the department  15 vector inspectors  1 secretary | 2 coordinators  20 fieldworkers  3 vector inspectors  1 secretary  1 administrative assistant |
| Total FTE | 17 | 23 |
| Communication | 4 communication experts | 2 communication experts  1 head of the department |
| Total FTE | 4 | 3 |
| Policy Department | 1 head of the department  1 lawyer  2 researchers  8 policy officers (4 for health care, 3 for public health, and 1 for veterinary public health) | 1 head of the department  1 medical doctor  1 statistician  1 health scientist  1 health promoter |
| Total FTE | 12 | 5 |

Adapted from Curaçao: Business plan, Ministry of Health, Environment and Nature (p. 80-98), by MoHEN, 2011. Adapted with permission.
